# Quantification of the Heterogeneity of Prognostic Cellular Biomarkers in Ewing Sarcoma Using Automated Image and Random Survival Forest Analysis

**DOI:** 10.1371/journal.pone.0107105

**Published:** 2014-09-22

**Authors:** Claudia Bühnemann, Simon Li, Haiyue Yu, Harriet Branford White, Karl L. Schäfer, Antonio Llombart-Bosch, Isidro Machado, Piero Picci, Pancras C. W. Hogendoorn, Nicholas A. Athanasou, J. Alison Noble, A. Bassim Hassan

**Affiliations:** 1 CR-UK, Tumour Growth Group, Oxford Molecular Pathology Institute, Sir William Dunn School of Pathology, University of Oxford, Oxford, United Kingdom; 2 Institute of Biomedical Engineering, Department of Engineering Science, Old Road Campus Research Building, University of Oxford, Headington, Oxford, United Kingdom; 3 Institute of Pathology, Heinrich-Heine University, Medical Faculty, Düsseldorf, Germany; 4 Pathology Department, University of Valencia, Valencia, Spain; 5 Research, The Rizzoli Institute, Bologna, Italy; 6 Department of Pathology, Leiden University Medical Center, Leiden, The Netherlands; 7 Nuffield Department of Orthopaedics, Rheumatology and Musculoskeletal Sciences, Nuffield Orthopaedic Centre, University of Oxford, Oxford, United Kingdom; University of Campinas, Brazil

## Abstract

Driven by genomic somatic variation, tumour tissues are typically heterogeneous, yet unbiased quantitative methods are rarely used to analyse heterogeneity at the protein level. Motivated by this problem, we developed automated image segmentation of images of multiple biomarkers in Ewing sarcoma to generate distributions of biomarkers between and within tumour cells. We further integrate high dimensional data with patient clinical outcomes utilising random survival forest (RSF) machine learning. Using material from cohorts of genetically diagnosed Ewing sarcoma with EWSR1 chromosomal translocations, confocal images of tissue microarrays were segmented with level sets and watershed algorithms. Each cell nucleus and cytoplasm were identified in relation to DAPI and CD99, respectively, and protein biomarkers (e.g. Ki67, pS6, Foxo3a, EGR1, MAPK) localised relative to nuclear and cytoplasmic regions of each cell in order to generate image feature distributions. The image distribution features were analysed with RSF in relation to known overall patient survival from three separate cohorts (185 informative cases). Variation in pre-analytical processing resulted in elimination of a high number of non-informative images that had poor DAPI localisation or biomarker preservation (67 cases, 36%). The distribution of image features for biomarkers in the remaining high quality material (118 cases, 10^4^ features per case) were analysed by RSF with feature selection, and performance assessed using internal cross-validation, rather than a separate validation cohort. A prognostic classifier for Ewing sarcoma with low cross-validation error rates (0.36) was comprised of multiple features, including the Ki67 proliferative marker and a sub-population of cells with low cytoplasmic/nuclear ratio of CD99. Through elimination of bias, the evaluation of high-dimensionality biomarker distribution within cell populations of a tumour using random forest analysis in quality controlled tumour material could be achieved. Such an automated and integrated methodology has potential application in the identification of prognostic classifiers based on tumour cell heterogeneity.

## Introduction

Tumours arise in somatic tissues and differ by site of origin, histological appearance and mechanistic hallmarks [1]. Inter-tumoural heterogeneity (between individuals) at the genomic, epigenomic and proteomic level, can be quantified by whole genome massively parallel (deep) sequencing and quantitative proteomics [2], [3]. Single cell sequencing data have recently also highlighted the significant intra-tumoural heterogeneity (between cells of a tumour), the fundamental clonal basis to the often rapid emergence of resistance to therapies [4]–[9]. Intra-tumoural heterogeneity is also modified by cellular and micro-environment context. Such contexts appear critical to tumour behaviour, but are generally less well understood, despite the evidence suggesting that tissue dependent niches established by stromal cell populations may substantially alter signalling responses and behaviours of tumour cells [4], [10]. In the clinical situation, patients are often stratified into different clinical cohorts. Further sub-classifications are either based on outcomes (e.g. survival), by genome wide analysis and the differential response to drugs, adding further heterogeneity at the clinical level [11], [12]. Despite the complex genomic heterogeneity between single cells in different regions within the same tumour, it remains unclear what the prognostic significance of these observations are without clinically applied and unbiased approaches to quantify associated biomarkers of cellular heterogeneity functional *in situ* [6], [13]. Moreover, ‘omics’ technologies generate large numbers of quantifiable features that greatly exceed the numbers of subjects in studies. Such high-dimensional data thus requires novel approaches to bio-informatics analysis. For cancer, one of the major general problems remains the unbiased integration of the high dimensional heterogeneity (distribution) data into predictive, prognostic and personalised tools that may have genuine clinical utility.

At the level of proteomic localisation in tissues, clinically useful tissue based proteomics is still largely based on localisation of protein antibody epitopes in formalin fixed material. For example, biomarker assessments using immunohistochemistry often remain semi-quantitative (+1, +2, +3), and lack the cellular localisation afforded by immunofluorescence and confocal imaging [14]. Use of MALDI based mass spectrometry [15]–[18], automated quantitative analysis (AQUA) [19] and multiple-ligand epitope cartography (MELC) [20], [21] offer significant technical advances, but they remain prone to bias and fail to address the quantification of heterogeneity at the resolution of each cell. Advances in unbiased image analysis, both in 2 and 3 dimensions, have facilitated the segmentation of regions and cells of tumours, and offer significant benefits in parallel with developments in fluorescent confocal microscopy. By high content image analysis, it is now possible to quantify biomarkers and their distribution in tumours, even though there are fundamental on-going problems with the interpretation of thresholds or ‘cutpoints’, in addition to the emerging quality control problems relating to tissue pre-analytical processing [19], [22]–[25]. Despite some of these technical limitations, the more basic application of virtual microscopy and image analysis to diagnostic pathology have lead to more widespread technology adoption, and so supports the impetus for the further development of novel computer algorthims, as for example those utilised in content retrieval [26], [27].

Here, we developed an integrated analysis pipeline to address the quantification of image feature heterogeneity with clinical prognostic outcome in a rare but genetically diagnosed tumour. Ewing sarcoma family of tumours (ES) are rare high-grade tumours of young adults with an incidence of 0.13 per 100,000 [28]. Diagnostic methodology developments have improved ES diagnoses, such that current standardisation also identifies the 20% of cases that arise in soft tissue rather than the more frequent primary site in bone. ES diagnosis is confirmed by an *in situ* hybridisation test based on the EWSR1 gene break-apart probes (EWS-FLI1, t(11;22)) with histological features of small round cells and anti-CD99 antibody cytoplasmic labelling [29]. In parallel with improved molecular diagnosis, combined modality treatment protocols have also been developed. These treatments incorporate dose intensive chemotherapy, surgery and radiotherapy and result in a 60–70% 5-year survival. Patient survival in ES correlates well with a number of clinical features, such as the good prognosis in small volume localised tumours with good histological response to primary treatment [29], [30]. There are no prognostic tissue biomarkers currently in use in ES that have been prospectively validated, even though ES provides an ideal starting point as these tumours correlate with a characteristic genotype. There is also remains a lack of biomarker related studies aiming to either stratify patients for standard treatments in the clinic, or to be incorporated into new prospective clinical trials or to be applied in experimental early phase protocols [31]–[33]. To date, the Ki67 proliferative biomarker has been shown to be of potential prognostic significance in some large series of ES patients using semi-quantitative single antibody immunohistochemistry [34]. Moreover, biomarker assays to potentially select the 5–15% of patients more likely to respond to novel IGF pathway intervention remain unavailable, with drug development programmes curtailed as a result [35], [36].

We sought to develop an unbiased tissue image segmentation algorithm to quantify downstream signaling biomarkers of receptor tyrosine kinase activation, detected with antibodies to signaling proteins and fluorescent probes, and imaged with multi-spectral confocal microscopy. Using this method to validate antibody probes firstly in ES cell lines, and then in ES tumour tissue, we obtained quantification of the distribution of biomarkers within the nucleus and cytoplasm of each cell within tumour biopsies assembled on a tissue microarray (TMA). We analysed the high dimensional distribution data in relation to patient overall survival using machine learning (random survival forest, RSF) and utilised the internal validation process in RSF to identify a prognostic classifier.

## Methods

### Cell culture

Cells were obtained from ATCC or different partner institutes of EuroBoNeT (Table S1) [37]. All EWSR1 translocation confirmed Ewing sarcoma (ES) cell lines used in this study were grown in RPMI 1640 (PAA Laboratories GmbH, Austria) supplemented with 1% Penicillin-Streptomycin (PAA Laboratories GmbH, Austria) and 10% Foetal Calf Serum (Biosera, UK). Three cell lines (STA-ET 2.1, STA-ET10, WE-68) needed to be cultivated in gelatine-coated culture flasks to allow cells to attach. For growth factor experiments cell lines were grown on coverslips (d = 13 mm) in 24-well plates (Costar, USA) with 4×10^4^ cells per well. Poorly attaching cell lines (e.g. STA-ET 2.1, STA-ET10, WE-68) were seeded on either Matrigel or gelatine coated coverslips (growth factor reduced, BD Biosciences, UK). After adaption for 2 days, cells were serum-starved in RPMI 1640 supplemented with 1% Penicillin-Streptomycin for 24 hr and treated with IGF2 (50 ng ml^−1^, R&D systems) for 1 hr at 37°C. Finally cells were fixed in 4% (v/v) formaldehyde for 15 min at room temperature (RT).

### Paraffin-embedded cell cores

Four Ewing cell lines (CHP-100, RD-ES, SK-N-MC, A673) were grown in petri-dishes (d = 14 cm) for 48 hr prior to 24 hr of serum starvation. After treatment with IGF2 (50 ng ml^−1^) for 1 hr, cells were trypsinised (TrypLE Express, Life Technologies, UK) and centrifuged at 1000 rpm for 5 min. Cells were re-suspended in 4% (v/v) formaldehyde in PBS, and fixed for 1 hr at RT. After centrifugation at 1300 rpm for 2 min, cells were re-suspended in 2% (v/v) molten agarose at 60°C (Hi-Res standard agarose, Geneflow, UK)/4% (v/v) formaldehyde in PBS, centrifuged at 2000 rpm for 20 sec and kept on ice for 30 min for the agar to set. The agarose block containing the cell pellet was dehydrated in an ascending alcohol series and embedded in paraffin. Five micro-meter slices were cut and transferred to Polysine slides. Slides were dried over night at 37°C and stored at 4°C until immune-labelling was performed within 7 days.

### Immunofluorescence

Cells on coverslips were washed in TBS for 5×3 min, permeabilised and blocked in TBS/0.5% (v/v) Triton X-100/10% (v/v) goat serum for 1 hr, and incubated with the primary antibody at 4°C overnight. Monoclonal rabbit antibodies pS6 (#4857), Foxo3a (#2497; #9467), EGR1 (#4154) and pMAPK (#4370) were purchased from New England Biolabs (UK). After 3 washing steps, cells were incubated with a secondary goat anti-rabbit antibody Alexa 594 (Life Technologies, UK) for 2 hr at RT. Fluorescent Phalloidin Alexa 488 (Life Technologies, UK) was used in order to detect the actin cytoskeleton. Cells were incubated with Phalloidin Alexa 488 (1∶50) for 2 hr at RT following 3×3 min washes in TBS. Nuclei were detected with the DNA stain DAPI (Sigma, UK) and coverslips were mounted with Prolong Gold Antifade (Life Technologies, UK). In order to determine the dilution of antibodies, we first serially diluted the secondary antibodies until the signals were at background level. We then titrated the primary antibodies in the same way, so that signal was detectable above background. More details for the validation of the antibody probes are in Table S2. Antibodies were further validated by use of inhibitors in cell lines (e.g. MCF-7). Reduction in either signal intensity or altered cellular distribution in response to IGF2 were confirmed by addition of inhibitors with a range of concentrations (maximal inhibitory concentration); rapamycin (10nM), LY294002 (10 µM) and U0126 (10 µM), for pS6 (signal reduction), Foxo3a (nuclear localization) and EGR1 (cytoplasmic localization), respectively.

### Tissue processing and image capture

Ewing sarcoma TMAs were derived from core biopsies obtained from the Department of Pathology, University of Valencia, the Institute of Pathology, University of Düsseldorf, Medical Faculty, and the Department of Pathology, Leiden University Medical Centre in collaboration with the EuroBoNeT biobank (Table S3). Written informed consent was obtained for all patient samples, and TMA cores coded with anonymised codes linked to clinical data that was also anonymised with the same code, with the latter data transferred with the physical TMA's as part of the EuroBoNET biobank. For tissue assembled into TMA from Valencia that included the Rizzoli Institute in Bologna, Italy, local consent was obtained for research on anonymised tumour material as part of the Prothets-503036 and EuroBoNeT-011814 bio-banks via the ethical committee of the Rizzoli Institute and Institutional Ethical Committee of Valencia University, respectively. For tissue assembled into TMA from Düsseldorf, informed consent was obtained for tissue for studies through the EUROPEAN Ewing Tumour Working Initiative of National Groups, Ewing Tumour Studies 1999 (EuroEwing99), in conjunction with institutional ethics committee of the University of Münster, Germany. For tissue assembled in the TMA from LUMC, all samples were handled according to the Dutch code of proper secondary use of human material as accorded by the Dutch society of pathology (www.federa.org). The samples were handled in a coded (pseud-anonymised) fashion according to the procedures as accorded by the LUMC ethical board. Importation of anonymised tissue was approved under UK HTA licence and local ethical review to the Sarcoma component of the Oxford Research Biobank (HTA 12217, Research Ethics Committee Oxford- C, REC 09/H0606/5). The HTA licence also covered informed consent for both importation of material and for project consent for biomarker studies of signalling proteins in sarcoma tissue.

A total of 524 patients were initially identified from three cohorts. 472 patients had TMA cores, but not all had outcome data. 314 potential biopsies were analysed with each TMA containing one (d = 2 mm, cohort a), three (d = 2 mm, cohort b) or two (d = 1 mm, cohort c) representative cores for each case, respectively. Of these, 52 cases had no cores listed on the TMAs obtained, and 77 had missing cores samples on slides (non-adherent) even though they had been incorporated into the TMAs. The remaining 185 cases had both visible cores on the TMA slides and associated outcome data and were therefore imaged. 5 µm thick TMA sections were dried for 10 min at RT before processing. After de-waxing and rehydrating, TMAs were permeabilised with TBS/0.5% (v/v) Tween20 for 30 min at RT, and washed several times in distilled water. For antigen de-masking, slides were immersed in citrate buffer (pH 6.0) and antigen retrieval was performed in a pressure cooker (Biocare Medical, UK) for 2 min at 125°C followed by 10 min at 85°C. Non-specific binding was blocked in TBS/Tween20 (0.5%. v/v) and 10% (v/v) goat serum for 1 hr at RT. Primary antibodies rabbit anti-Ki67 (1∶300, Thermo Scientific, UK), rabbit anti-Egr1 (1∶50), rabbit anti-Foxo3a (1∶100) and rabbit anti-pMAPK (1∶50) (New England Biolabs, UK) were incubated simultaneously with mouse anti-CD99 (1∶100, Leica, UK) in a humidified chamber at 4°C over night. After washing in TBS, both secondary antibodies (goat anti-rabbit Alexa 488 and goat anti-mouse Alexa 555, Life Technologies, UK) were added together for 2 hr at RT. Slides were washed and blocked with TBS/10% (v/v) rabbit serum for 1 hour before incubation with the primary conjugate pS6 Alexa 647 (1∶30, New England Biolabs, UK, #4851) for 2 hr at RT. Nuclei were labelled with DAPI and slides were mounted with Prolong Gold Antifade (Life Technologies, UK). Images were acquired with an Olympus Fluoview FV1000 confocal microscope and a 60x oil objective (NA: 1.35). Images of 2048×2048 pixels, had a horizontal and vertical dimension of 211 µm×211 µm and a thickness of 1.292 µm. Between 1 and 6 images per patient were captured depending on the size of the tissue core. Median filtering and Gaussian smoothing were applied to all images to reduce image noise. The total processing of samples to final images took on average 2-3 days per TMA slide.

### Image analysis

Segmentation: To segment individual DAPI stained nuclei, Otsu's method was used to initialise a level set algorithm [38]. A hybrid geodesic region-based level set was used to estimate location and shape of individual nuclei [39]. This estimate was corrected using a watershed tesselation for cells in tissues in order to divide clumps of nuclei. For the cytoplasmic segmentation, thresholding was used to find the outer boundaries of cells. A Voronoi tesselation was first calculated based on an equidistant partitioning between neighbouring cells [40]. The cytoplasm of cells in cores were segmented firstly with a watershed tesselation, followed by an iterative marker controlled watershed method to find the inner boundaries based on the intensity gradient of CD99. This cytoplasmic segmentation algorithm was then applied to tissue sections. Nucleus and cytoplasm segmentations were validated by comparing the computed segmentation results with manual results drawn by 3 human experts. For this purpose, images from 3 different cell lines (A673, SK-N-MC, RD-ES) on coverslips and as cell cores were analysed. The results of the validation are shown as Bland-Altman plots, which compare the areas of all nuclei found by 3 experts to the areas according to the segmentation algorithm, and plots of the Hausdoff distance, which is the size of the worst mismatch between both segmentation results. *OxBioPathv1* integrates the segmentation and analysis (below) and is written in Matlab. The total time to process automated segmentation of all images took 24–48 hours using University of Oxford computing services.

CD99 and Ki67 thresholding: A Ki67 index for each patient was defined as the proportion of CD99 positive cells that were Ki67 positive, as defined by thresholding the log2 (nucleus/cytoplasmic ratio) of each marker. For the RSF analysis CD99 positive cells were defined as those with mean cytoplasmic CD99> mean nuclear CD99.

Principal Component Analysis (PCA): PCA is a dimensionality reduction tool which allows an initial visualisation of the data to be created in two or three dimensions, by searching for linear combinations of features corresponding to the modes of maximum variation in the data. Z-scores were calculated to transform all features to a common scale (mean 0, variance 1) before the PCA calculation.

Patient features: Individual cell features from all images associated with a patient were summarised by using kernel density estimation to approximate the probability density function (PDF) of each feature. The PDF for each feature was evaluated at 100 equally spaced points.

Automated Quality Control: Image features were only calculated for those images which passed the quality control criteria, which primarily related to the numbers and proportion of cells in focus. In order to support future automation of the entire pipeline, we built an automated classifier using a set of image features designed for high content screening (HCS) using the DAPI channel from images also stained for Ki67 (916 images). 230 of these images were labelled as poor quality and 686 as good quality. Four HCS QC features were calculated for each image [41]:

1. Inverse coefficient of variation: Mean image intensity/Standard deviation image intensity
2. Focus score: Variance image intensity/Mean image intensity
3. Image correlation: A measure of the correlation between neighbouring pixels in an image, calculated from the grey-level co-occurrence matrix (GLCM) [42]. Image intensities were quantised into eight levels, and the GLCM was calculated at a single scale for neighbouring pixels.
4. Power log-log slope (PLLS, across the whole range): The change in the intensity power spectrum of an image is described as a function of spatial frequency. The 2-dimensional FFT of an image was calculated, and log10 (squared magnitude of FFT) plotted against log10 (spatial frequency), ignoring orientation. The gradient of this plot was calculated across the full spatial frequency range.

An additional eight features were obtained by splitting the power log-log plot into eight spatial frequency ranges, before calculating the slope for each. This corresponds to the PLLS at different image scales. These features were used to train two logistic regression classifiers to distinguish between good and poor quality images, one using the first four features (x1-x4), and the other using all twelve features (x1-x12). The performance was evaluated using leave-one-out cross-validation.

Random Survival Forest: The RSF training process involved building a set of decision trees from a subset of the original dataset, using sampling with replacement (bagging). Since each tree was built from a different subset of samples the remaining out-of-bag (OOB) samples provided an unbiased estimate of the error rate, calculated using Harrell's concordance index [43], so that 0 indicated a perfect prediction whereas 0.5 would be expected by chance. At each step during the construction of each tree a random subset of features was tested for predictive capability. This ensured all trees were different, contributing to the resilience of RSF to over-fitting.

In common with most machine learning algorithms, the performance of RSF was improved by using a feature selection algorithm to discard irrelevant features. We used the variable hunting algorithm included in the RSF package, which introduced an additional random partitioning of the data (80% train and 20% test) before the forest was trained. Features were iteratively introduced until no significant features remained, after which the process was repeated multiple times with a different sampling of the data. Since each iteration was independent of all others the importance of a feature could be measured by how frequently it was selected.

In addition the internal RSF error rates were further validated using cross-validation in which the data set was randomly partitioned into a training set consisting of two-thirds of the samples on which the RSF was trained, with the remaining one-third of samples used for testing. This was repeated 50 times for each set of features. Cross-validation is a valuable tool for analysing the expected performance of the algorithm since a single analysis may lead to an apparently well (or poorly) performing algorithm by chance, whereas the use of multiple resamplings mimics the analysis of multiple different datasets.

RSF parameters:

Number of trees: 1000

Splitting rule: Logrank random (default)

Number of features tested at each split: N/3 (default).

## Results

### Cell line segmentation

**Figure 1:**
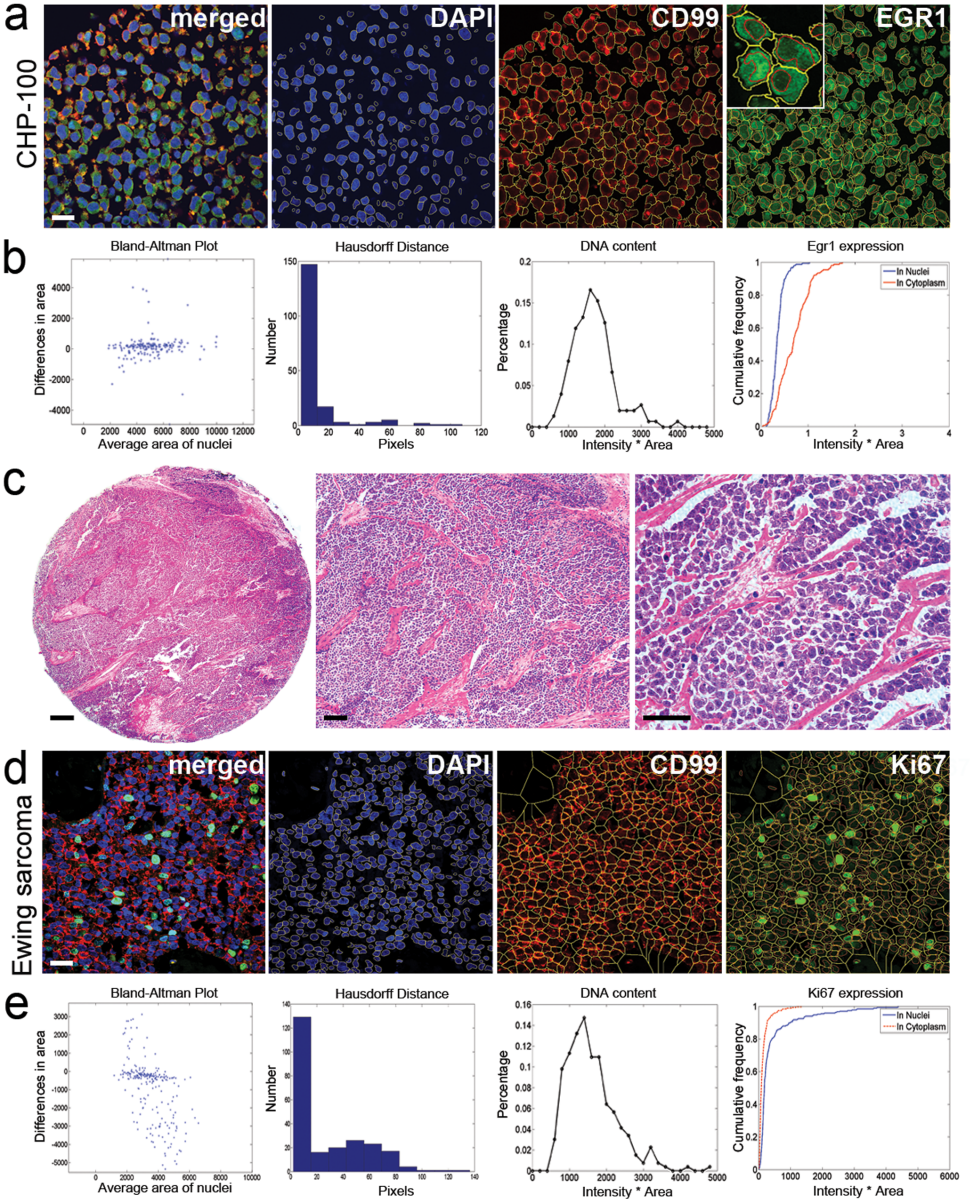
Development of a single cell segmentation algorithm for tumour tissues. Confocal images of agar pellets of EWS-FLI1 positive Ewing sarcoma cell lines were used to optimise image segmentation. **a**. Multi-channel and single channel images (with segmentation lines) of the CHP-100 cell line in cores labelled with DAPI, CD99 and EGR1 biomarkers indicating nucleus and cytoplasm localisation, respectively (see high magnification insert). In **b**., >500 cells were manually segmented and compared to the image segmentation algorithm (see Fig. S1 in File S1) using Bland-Altman and Hausdorff distance. Also, example distributions are shown for nuclear DNA content and nuclear and cytoplasmic localisation of EGR1. Image segmentation was applied to tissue microarrays of **c**. Ewing sarcoma core biopsies on a tissue microarray (TMA), and **d**., multi-channel confocal images captured for DAPI, CD99 and Ki67 proliferation marker. In **e**. >500 cells from TMA cores were manually segmented and compared to the image segmentation algorithm using Bland-Altman and Hausdorff distance, with distributions for DNA and Ki67. Bars: 20 µm (**a**. and **d**.), and in **c**, 200, 100 and 50 µm, respectively.

We first obtained imaging data of biomarker localisation and distributions using unbiased image segmentation of cell lines and cell line cores. A panel of 10 validated ES cell lines (Table S1) were exposed to serum free culture and to the growth factor ligand, insulin-like growth factor 2 (IGF2). A novel image segmentation algorithm (see methods) was used to identify the boundaries of the nucleus and cytoplasm of each cell (Figure S1 and S2 in File S1). Simultaneous fluorescent detection of Phalloidin, DAPI and signaling biomarkers such as pS6, Foxo3a or EGR1, were imaged by multi-spectral confocal microscopy (Figure S2 in File S1). Segmented images were validated relative to three experts using Hausdorff (maximum discrepancy between boundaries) and Bland-Altman plots of segmented areas (Figure S2b, d, f in File S1). Cumulative frequency plots revealed cell line specific effects of biomarker distributions within the nucleus and cytoplasm of each cell in response to IGF2, indicating there was significant heterogeneity between each cell's response within a given cell culture, and that the biomarker antibodies were informative for response (Figure S3 in File S1). An improved version of the segmentation algorithm was then validated using the same cell lines assembled into cell pellets, where there were frequent associated clumps and overlapping cells that more closely resembled tumours (Figure 1a, b and Figure S4 in File S1).

### Tissue micro-array segmentation

Image segmentation of Ewing sarcoma tissue biopsy cores from three separate but similarly treated ES clinical cohorts (Table 1) assembled TMAs (Figure 1c, d) were next validated using Bland-Altman and Hausdorff plots (Figure 1e). The accuracies of the image segmentations in cell culture, cell pellets and tumours were relatively similar, although overlapping cells and the disrupted cell architecture in tumours presented more challenges to the final segmentation algorithm. The number of cells segmented per image was in the range 50–500 depending on cell density, and up to 4 images were captured from random sites on each TMA core. We utilised TMAs where cores were present on slides linked to clinical outcome data for 185 ES cases confirmed by EWSR1 break apart *in situ* hybridisation probes sourced from the three separate patient European cohorts (a = 57, b = 20, c = 108). Following imaging of all TMA cores labelled with DAPI, CD99 and pS6, we then applied strict image based quality control criteria in order to discard non-informative images, including those where nuclei could not be segmented using DAPI (Figure S5 in File S1). Image normalisation included controlling for image bias of the confocal microscope, image pre-processing and comparison of feature values between cohorts. As a result of the quality control based on these criteria for each image, 36% of the sampled images had to eliminated because of poor quality (67/185), leaving a total of 118 cases with truly informative images suitable for further analysis (Table 1, a = 43, b = 16, c = 59).

**Table 1:** Clinical features of patients with informative images from cohorts utilised in combined random forest analyses.

Ewing sarcoma clinical features	Cohort a (n = 43)	Cohort b (n = 16)	Cohort c (n = 59)
Status (% alive)	70	25	54
Male: Female ratio	1.26∶1	3∶1	1.23∶1
Mean age	19	19	18
Site (%, central: peripheral: extra-osseous)	39∶33∶28*	19∶75∶6	24∶76∶0
Tumour volume (%, <200 ml: ≥200 ml: ND)	63∶25∶12	13∶25∶62	NA
Metastasis (% Yes: No: ND)	98∶0∶2	69∶19∶12	34∶66
Translocation (%, EWS-FLI1: Non-FLI1#: ND)	77∶9∶14	31∶38∶31	88∶0∶12

*ND cohort a  =  outside bone

# Non EWS-FLI1  =  EWS-ERG; EWS-NFATC2 or EWSR1-re-arranged (no FLI or ERG partner).

Note: all patients treated in Europe with standard chemotherapeutic, radiotherapy and surgical trial protocol (EICESS92/EE99 CESS81).

ND: data not available.

The elimination of a significant number of samples as a result of quality control indicates that sample pre-analytical processing is a key and independent determinant of the utility of biomarker analysis. The formalin fixed samples assembled into paraffin blocks were processed by local laboratories in the different cohorts. There were no systematic records collected for the processing and storage of the blocks and only local guidelines stipulated for the diagnostic samples. The primary core blocks were sampled and assembled in the TMAs specific for each cohort. We assume that a large source of variation relates to timing of tissue collection and fixation, the length, type and conditions of storage of samples as all such pre-analytical steps contribute to variations in sample quality and biomarker stability.

The Kaplan Meier overall survival analysis confirmed that the patient outcome was different between cohorts (Figure S6 in File S1). These differences were due to different relative proportions of patients with relatively good or bad prognosis in each cohort. Importantly, the informative cases that were imaged were however representative of the total for all cohorts combined (passed and failed), and with respect to the different survival outcomes per cohort (Figure S6 in File S1). We tried to confirm our use of manual criteria to select images that could be informative by building a logistic regression classifier for quality control of high content imaging using the passed and failed images. In the two classifiers tested, we failed to clearly identify the passed and failed images, with the AUC for ROC curves both being 0.72 (Figure S7 in File S1). Thus the images of the samples that had not passed quality control could yet be informative in terms of clinical outcome data, but it remains necessary to develop quality control classifiers in the future to select regions in tumours that can be analysed.

Within the informative imaged data, significant differences still existed within each cohort with respect to principle component analysis (PCA), in line with the differences in patient survival, although as expected TMA cores for the same patient imaged separately segregated together and were found to be relatively consistent (Figure S8 in File S1). The patient samples within each cohort appeared heterogeneous in terms of outcome data as above, reflecting not only the differences in survival in each cohort, but also suggesting the importance of normalised pre-analytic processing to reduce data variation [44]. Overall, 476 multi-spectral registered confocal images were captured from material from 118 patients and resulted in the segmentation of a total of 113,201 individual cells with an average of four biomarkers per cell (10^4^ features per patient). Each biomarker was associated with a cellular distribution (in relation to the nucleus-DAPI and cytoplasm-CD99 segmentation) in each cell. Although the remaining 118 imaged cases did not differ in survival from the combined cohort, the final number of patients would normally severely limit the potential for a conventional training and validation approach to the characterisation of single biomarkers using the separately identified cohorts. In addition, as others have found, our data undermines the assumption of the REMARK guidance, that non-normalised data from separate cohorts are similar enough to make experimentally valid attempts at conventional cross-validation of imaging biomarkers [45]. Moreover, the high-dimensionality of our data set compared to the size of the patient cohorts even if we had included all patients, would have still remained the main challenge to any subsequent analysis. This would have remained the case even if several hundred high quality validation samples could have been identified. Table S3 lists the anonymised patient data with respect to imaged data. Raw images and software can be freely downloaded; http://www.ibme.ox.ac.uk/research/biomedia/software-and-datasets.

### Conventional biomarker analysis

**Figure 2:**
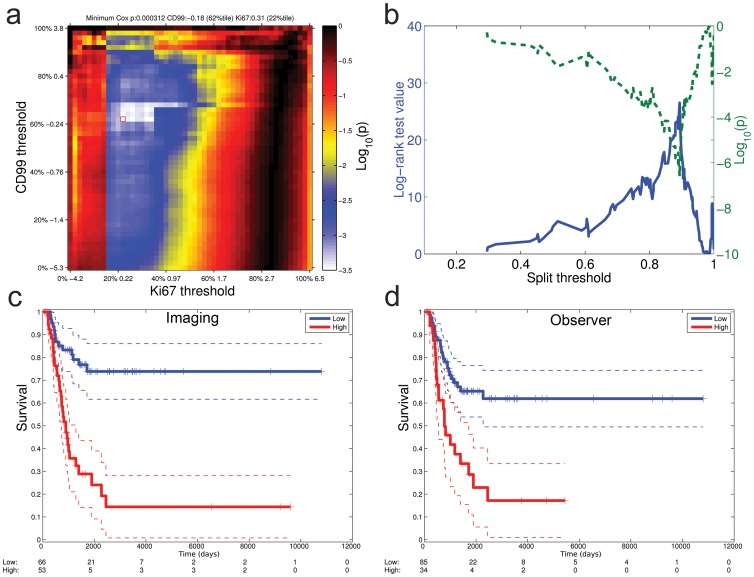
Image thresholds bias significance of tissue biomarker results. **a**. Thresholds for CD99 and Ki67 were split into centiles of the log_2_ (nuclear/cytoplasmic ratio) distribution. The heat map shows the Cox regression *p* value (log_10_) for each pair of centiles with respect to survival outcome of the whole imaged cohort. The square marker shows the optimal threshold with respect to the *p* value (CD99; −0.18 [62%tile], Ki67; 0.31 [22%tile]. **b**. Using this pair of thresholds, the Ki67 index and the log-rank test is calculated for all possible dichotomised splits of patient groups. **c**. Optimal threshold values defined in **a**. and **b**. lead to the Kaplan-Meier plot of overall survival outcome for good (low Ki67 index, blue) and poor prognosis (high Ki67 index, red) cases (dotted lines; 95% confidence intervals). Cox regression (b = 1.6, z = 4.8, p = 1.6×10^−6^, log-rank p = 2.6×10^−7^). **d**. A single observer (CB) scored the same images for Ki67 labelling by eye based on a binary low (blue) or high (red) score, resulting in the Kaplan Meier plot (Cox regression  = 1.3, z = 3.7, p = 2.5×10^−4^, log-rank p = 1×10^−4^).

In order to analyse the pooled image distribution feature data, we first undertook a conventional threshold approach. By combining an exhaustive range of thresholds (cut-points) with respect to the labels Ki67 (the proliferative marker) and CD99 (the cytoplasmic marker) with overall patient survival, a weighted regression accounted for non-linear dependence between total absolute intensity and intensity ratios using informative images (Figure S2 in File S1). We plotted Cox proportional hazards *p* values of the survival functions for each threshold setting on a heat map (Figure 2a). Using the maximal significance threshold (annotated in Figure 2a), log rank significance values were then also plotted for all possible splits (cut-points) of patients into two groups (Figure 2b). The data for the maximal significance value was utilised to generate the Kaplan-Meier plot (Figure 2c). By taking the same set of images, one experienced observer (CB) also screened the same images independently of the machine derived cut point and scored them using a visual threshold of whether samples showed subjectively high or low proportion of Ki67 labelling. The results showed that the Kaplan-Meier plots from the maximum threshold cut-point determined systematically, and the subjective decision of the independent observer appeared surprisingly similar, although not identical (Figure 2c and 2d). These data suggest that there are significant risks of bias when taking a subjective threshold value based on an observer, but also an inherent risk of setting incorrect thresholds if applied indiscriminately by a conventional image analysis to all cells in the image, and setting an arbitrarily determined threshold or cut-point. Both approaches have bias in that either might obscure or enhance the predictive role of a biomarker, for instance due to multiple comparisons leading to a significant result by chance.

### Random survival forest analysis

An alternative analysis was undertaken with an unbiased machine learning tool random survival forest (RSF). RSF was applied to the combined cohort of image features and associated overall survival data. Random forests (RF) and variants are powerful machine learning tools to automatically generate predictions from a dataset by combining multiple features, each of which may have a highly skewed non-linear distribution [46]. Importantly, RF can take into account both interactions and dependencies between features without them being explicitly encoded. Unsupervised analysis can also result in a form of clustering analysis, and this basic form of RF has been successfully applied to immuno-histochemistry based classification [47]. RSF is a specific supervised random forest variant recently developed that allows integration of multiple independent features to generate a predictive tool with respect to patient outcome (prognosis) in the form of time dependent survival data, without requiring the user to specify thresholds or cut-points [48], [49]. Importantly, the use of randomised ‘leave out’ and random feature selection reduce the problems of over-fitting inherent in many machine learning algorithms, particularly in our case with low sample numbers, and the lack of independent high quality validation patient sets.

**Figure 3:**
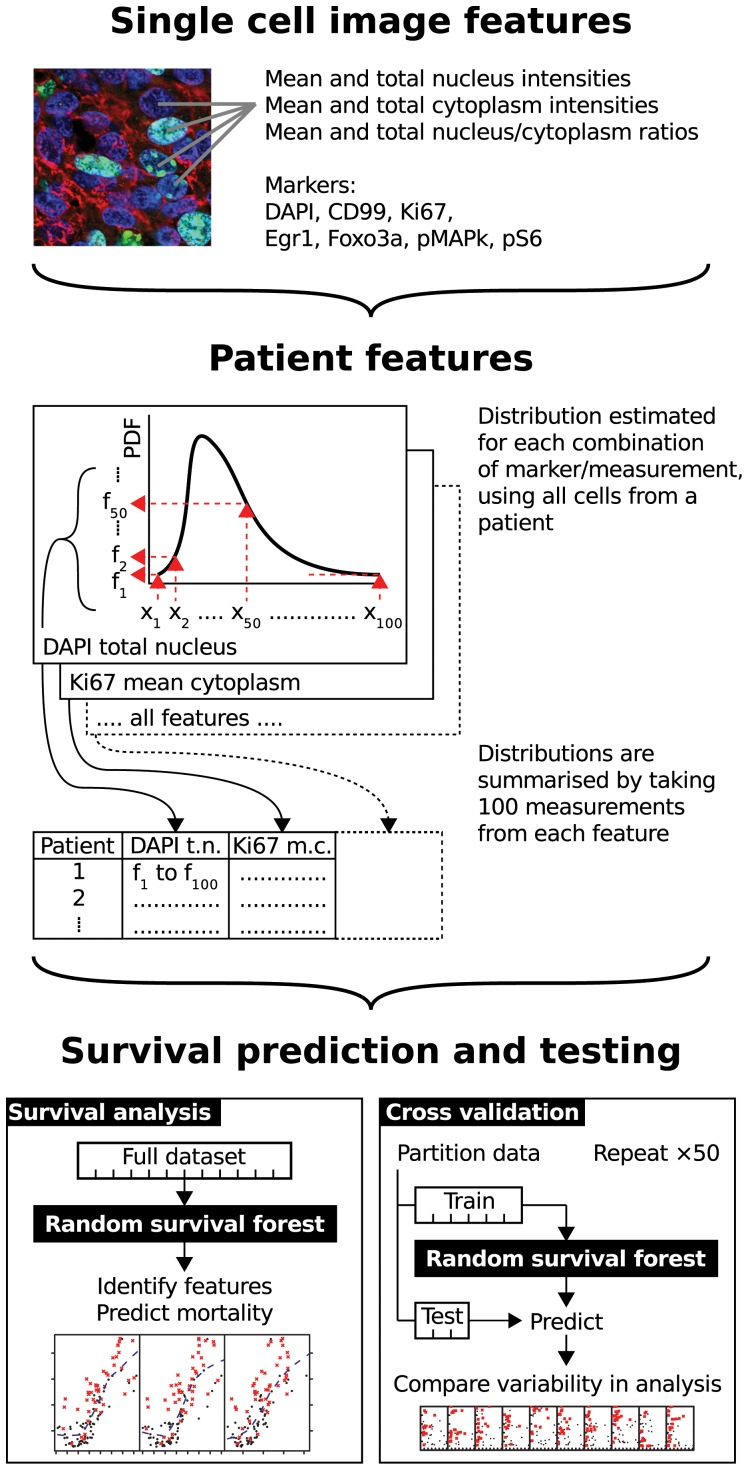
Random survival forest analysis of biomarker image feature distributions. An overview of the imaging, the RSF survival analysis algorithm and validation approach. Single cell features are combined into patient features by estimating the probability distribution (PDF) for each feature, and taking measurements of each distribution at 100 points. Each RSF is used to analyse all patients, with prognostic features identified. The use of bagging in each RSF means error rate estimates should be unbiased, and this is verified using randomised cross-validation. This procedure also allows the variability in performance of the algorithm to be simulated without requiring an additional dataset.

**Figure 4:**
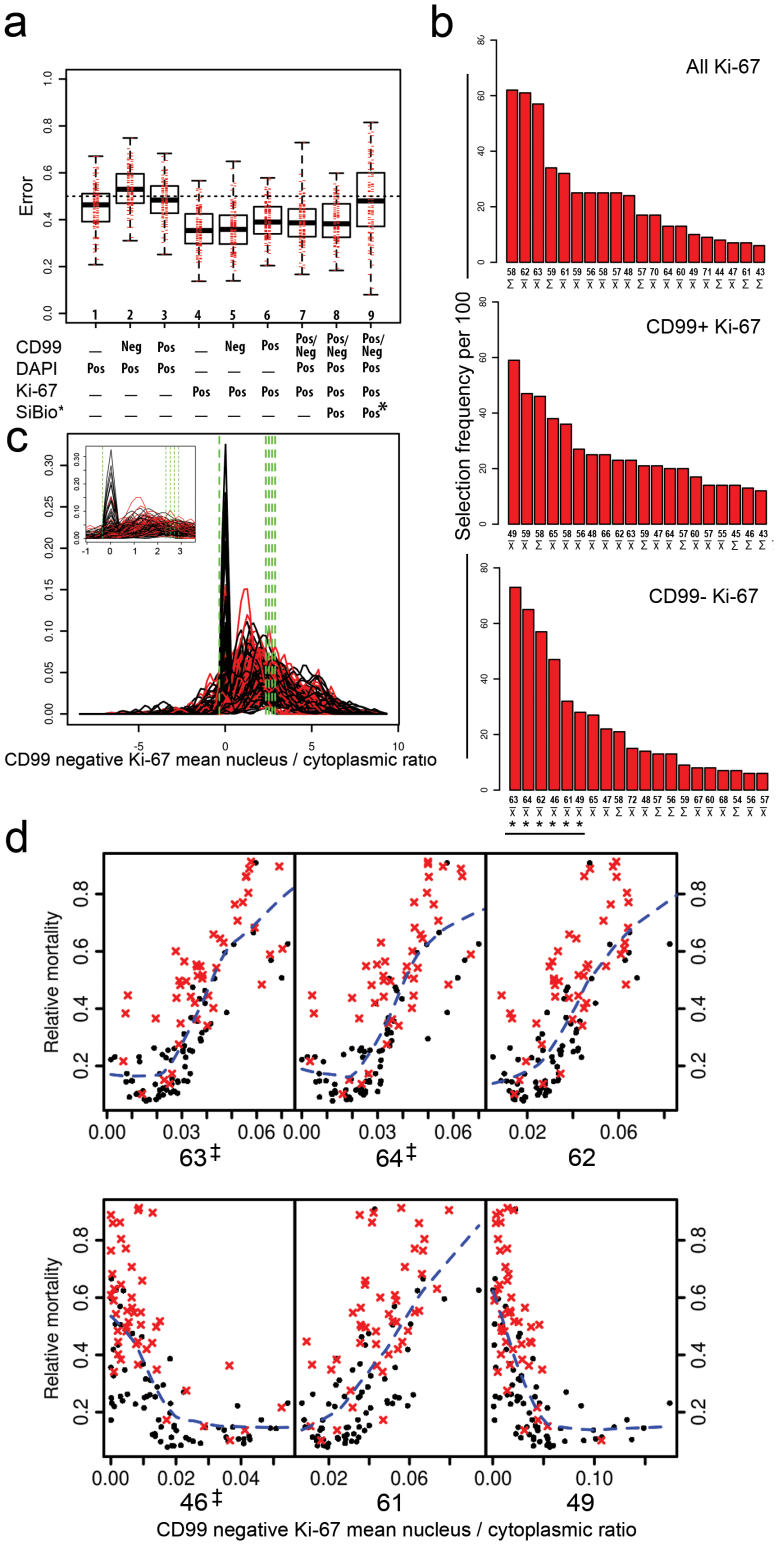
Random survival forest classifier error rates, distribution features and mortality. **a**. Error rates for nine RSF analyses trained with the variable hunting algorithm, shown as box plots (median line, inter-quartile range box, minimum and maximum). SiBio refers to combined analysis of signalling biomarkers Egr1, Foxo3a, pS6 with and without pMAPK*. Errors were lower for Ki-67 marker. See also Table 2. **b**. As each iteration of variable hunting is independent, so the frequency of selection of each feature and its overall ranking can be shown following 100 re-samplings. **c**. Selected features plotted (vertical lines) against the original distribution. Red and black lines indicate deceased and censored patients, with insert showing magnified plots. **d**. Based on 100 iterations of variable hunting RSF, an overall mortality plot can be generated as a function of the RSF and each feature. The top six (*) separate features are shown for the CD99 negative Ki-67 RSF, with the RSF integrating all ranked features**^‡^** within one classifier. Red crosses and black dots, deceased and censored patients, respectively.

Nine RSFs were performed (Table 2) and trained on the patient distribution features obtained from single cell features using the iterative variable hunting (VH) feature selection algorithm to identify features which were predictive of patient outcome, with overall cross-validation error rate measured using Harrell's concordance index (Figure 3) [43]. Instead of calculating a single error rate, which might be abnormally high or low due to chance, we obtained a distribution of error rates from the multiple iterations (Figure 4a), giving a more realistic view of the performance of the algorithm. Error rates were lower (<4.0) in the RSF with Ki67, Ki67 combined with DAPI and Ki67 combined with EGR1, Foxo3a and pS6 markers (Table 2, Figure 4a). Significant features were identified by comparing the number of times a feature was selected for the final model out of the 100 random VH iterations. For example, the ‘CD99 negative Ki67' RSF selected the feature ‘CD99 negative Ki67 mean nuclear/cytoplasm ratio' at the 63rd centile a total of 73 times (Figure 4b). The distribution of each patient's features for CD99 negative Ki67 mean nuclear/cytoplasmic ratio are shown (insert higher magnification, Figure 4c), along with the final five selected features marked by green dashed lines. Each feature of the classifier, although normally combined together, is also shown as single features compared to relative mortality (Figure 4d). Note that this shows the relative contribution to the RSF prediction of that feature, and importantly does not necessarily mean that the single feature could be used as a predictor on its own.

**Table 2:** Image feature distributions utilised in nine independent random survival forest analyses.

Random Survival Forest	Bio-Marker distributions	N	All features	Variable hunting		VH Rand-CV	
				mean	stdev	mean	stdev
1	DAPI^1^	118	0.54	0.45	0.093	0.49	0.055
2	CD99- DAPI^2^	118	0.57	0.53	0.086	0.54	0.064
3	CD99+ DAPI^3^	118	0.55	0.48	0.087	0.50	0.083
4	Ki67^4^	118	0.43	0.36	0.085	0.38	0.064
5	CD99- Ki67^5^	118	0.40	0.36	0.095	0.38	0.073
6	CD99+ Ki67^6^	118	0.43	0.40	0.080	0.41	0.065
7	CD99± DAPI Ki67^7, 8, 9^	118	0.44	0.39	0.092	0.39	0.062
8	CD99± DAPI Ki67 Egr1 Foxo3a pS6^7, 8, 9^	114	0.50	0.39	0.089	0.40	0.062
9	CD99± DAPI Ki67 Egr1 Foxo3a pS6 pMAPK^7, 8, 9^	50	0.42	0.48	0.158	0.48	0.104

1 DAPI: Nuclear DAPI (totals and means) in all cells.

2 CD99-DAPI: Nuclear DAPI in CD99- cells.

3 CD99+DAPI: Nuclear DAPI in CD99+ cells.

4 Ki67: Ki67 nuclear: cytoplasm ratios (totals and means) in all cells.

5 CD99-Ki67: Ki67 in CD99- cells.

6 CD99+Ki67: Ki67 in CD99+ cells

7, 8, 9 CD99+ Multiple markers in CD99+ and CD99- cells.

N: number of patients. Some patients did not have a full set of labelled images, so the number of samples used for each RSF varies depending on the combination of markers used. One patient did not have any cells labelled with Ki67. All features: the out-of-bag error rate calculated on a classifier containing all features from the indicated marker distributions. Variable hunting: the internal test set error rate using a RSF trained using variable hunting feature selection, and are the mean and standard deviations of the error rates are shown.

VH Rand-CV: the mean and the standard deviation of the cross-validation error rate using a RSF trained using variable hunting feature selection, with 50 rounds of two-thirds train: one-third test sets.

**Figure 5:**
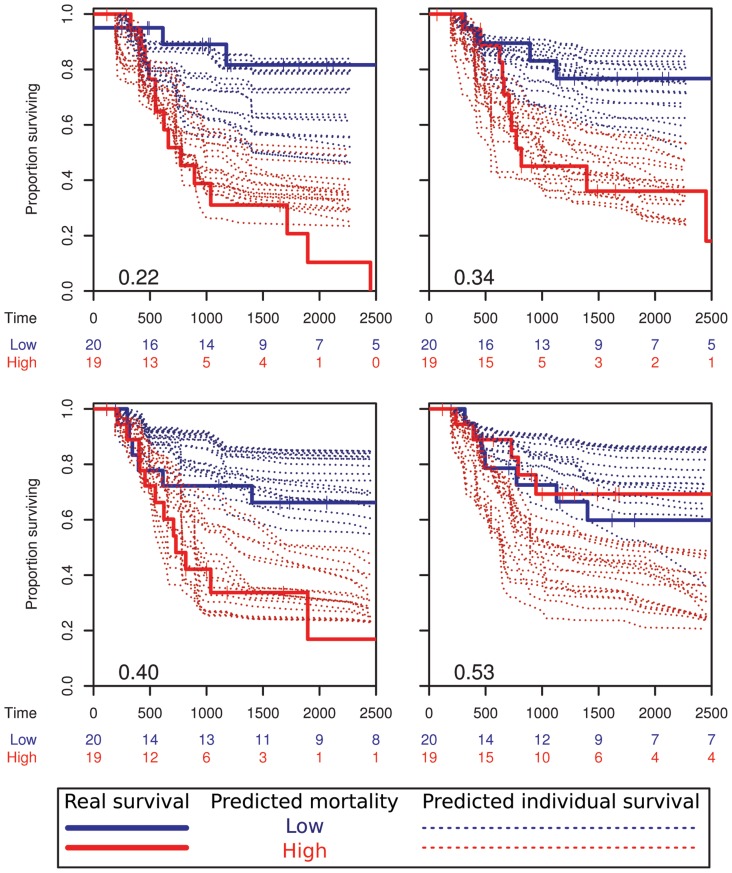
Cross-validation of the full RSF algorithm. The imaging dataset was randomly partitioned into a training set (e.g. two-thirds of patients  = 79) and a testing set (one-third of patients  = 39), with 50 repeats. Example results from 4 of the 50 cross-validation repeats for the CD99- Ki67 RSF, encompassing the full range of error rates (0.22–0.53) shown in black). Patients in the test set were divided into two approximately equal groups using the RSF predicted mortality (low, high) and survival curves plotted using the known survival data (solid lines), with a low error rate corresponding to a difference in survival of the groups. In addition to predicting mortality the RSF could also predict an individual time dependent survival curve for each patient (dashed lines). Plots are truncated at 2500 days, in some cases predicted survival times do not reach this far since they are limited by the last event in the randomly selected training set. (see also Figure S12 in File S1).

The internally generated RSF error rates should be unbiased, and to further validate this we used randomised cross-validation which, as expected, showed error rates consistent with this (variable hunting cross-validation, Table 2). This also enabled us to better understand the variation in performance by visualising the change in predicted output, as summarised by four of the cross-validation predictions for the CD99 negative (low cytoplasmic labelling) Ki67 RSF (Figure 5). Predicted mortality and survival plots for the test sets from 25 of all 50 partitions ranked by error rate are also shown (Figure S10, S11 and S12 in File S1).

Ki67 has been proposed as a prognostic biomarker for Ewing sarcoma, although here we specifically identified a sub-group of cells that were Ki67 positive but relatively CD99 negative, that is the nuclear/cytoplasmic ratio of the CD99 marker was less than 1 (Figure S9 in File S1). Following this result, we were able to specifically identify this population of cells in images that may have been overlooked using single biomarker analysis (Figure S13 in File S1). The biological basis of this top ranked feature, and the potential for an undifferentiated sub-population that it may represent, remains unknown. For example, the identification of CD133 positive stem cells would still require further experimental investigation [50]–[52]. Although the RSF classifier predicted mortality, the predictions for survival outcome were also consistent, and demonstrate the validity of the RSF classifier method (Figure S10, S11 and S12 in File S1). Importantly, each patient's predicted survival could be modelled, leading to a personalised prediction and risk stratification that fully incorporates heterogeneity of that patient's feature distributions.

## Discussion

We have been motivated to develop an unbiased analysis pipeline that is directed at the cellular distribution of biomarkers in tumours because of the biological problem of cell heterogeneity. Data integration strategies aimed at quantification of the heterogeneity of cells within a tumour are relatively under-developed, and may be limited by current multi-variate approaches. Automated methods for unbiased quantification of images have also been applied, yet frequently lack resolution at the individual cell, making it difficult to infer whether distributions (heterogeneity) between cells was evident [53]. Processing of high dimensional biomarker data has however been improved by application of machine learning algorithms, such as random forests, and so provide an important platform whereby informative components of data have the potential to be incorporated into a multi-parameter classifier [54].

Whilst the use of image segmentation in tissue sections has been widely applied, the analysis has largely been restricted to segmentation methodology, rather than to a complete integration with subsequent biomarker analysis in a clinically characterised sample set [55]–[57]. We validated known methods of image segmentation following capture of high resolution fluorescent confocal images from genetically defined tumour cell lines and tumour tissue [58]. The distributions of simultaneously captured fluorescent biomarkers within each cell of image were obtained using cytoplasmic and nuclear masks, and these were used to define boundaries between each cell. Here image segmentation was performed using two steps. The first used the well characterised local initiated thresholds using ‘level sets’ to define nuclear boundaries, which was then combined with a cytoplasmic marker iterative watershed method to segment cytoplasmic and cell boundaries. The distribution of image features within each cell and between all cells segmented in the image represents tumour cell heterogeneity. With the number of probes and images, we quickly generated high dimensional data per patient. Importantly, we incorporated information on heterogeneity by splitting the distribution of each biomarker (probability density function) in 100 equal segments (Figure 3). Importantly, by this novel approach, we captured the distributions of these features, and incorporated all the data into a random survival forest (RSF) tool to generate a prognostic classifier. When integrated analysis combining image analysis with biomarker evaluation has been performed, for example in the analysis of breast cancer stroma and in a RSF analysis in renal cell carcinoma, clinically significant findings were frequently generated when combined with methodology development [45], [59]. These precedents lead us to apply our unbiased methodology pipeline that integrated image acquisition, image segmentation and machine learning with RSF, to the discovery a clinical prognostic classifier.

In order to maximise the potential of the pipeline we needed to attempt to minimise variation in a test example. Variation includes fundamental differences in tumour cells attributed to different driver mutations, and to the differences in clinical outcome attributed to non-standardised diagnosis, stage of disease and treatment modalities. By sourcing cohorts of Ewing sarcoma biopsy material associated with standardised clinical management and outcome data, we attempted to enrich for molecular and clinical homogeneity, such that differences in outcome may be more likely to be attributed to differences in tumour behaviour sampled by the biomarkers that we quantified. Our initial observations indicated that there was considerable biomarker variation between different patients, but this was mainly in the quality of tissue material available for analysis, such that the potential of what initially appeared a unique combined cohort was significantly limited to samples that could actually be reliably analysed by the pipeline (informative material). This was an important observation and similar to those reported by others, especially in TMA assembled formalin fixed material [25], [60], [61]. Moreover, such observations undermine any analysis of separately collected and stored tissues under variable pre-analytic processing conditions, as combining these data sets are likely to compound either biased high content or even standard histological scoring systems using immuno-histochemistry. For future prospective validation, improved tissue ischaemic time and optimal preservation, such as with combined paraffin coating and nitrogen storage, will likely be mandatory [44], [62], [63].

Despite the limitation with material quality, the informative and high quality material lead to the identification of biomarker heterogeneity between each cell of a Ewing tumour core biopsy, including in the commonly assayed signalling pathways frequently deranged in these tumours, namely the MAPK and IGF-PI3K pathways. Our unbiased pipeline with RSF verified Ki67 as a potentially informative prognostic biomarker in terms of patient survival, but only it appears in a subset of cells with lower CD99 labelling than in the total cell population. It is known that cellular hierarchies exist in tumours, exemplified by rapid proliferative cell types (Ki67 positive), and concept of the cancer stem cell or tumour progenitor cells, that initiate and sustain tumour growth. For example, CD133 positive ES cells have been isolated that can sustain tumour growth through serial transplantation, and can differentiate into other lineages such as adipocytes and osteogenic cells [52], [64]. Moreover, mir145 and SOX2 are regulated by EWS-FLI1, and TARBP2 dependent miRNA maturation appears to be a major regulatory determinant of the cancer stem cells in Ewing sarcoma [65], [66]. Further hypothesis testing is required, as it remains unknown if the CD99 distribution in such progenitor cells is more variable and whether these cells are proliferative and so relate in any way to our observations.

As we also demonstrate, biomarker classifiers based on arbitrary cut-points of the imaged data could result in bias, a factor that remains a significant impediment to current techniques [14], [22]. These findings underscore the need for further unbiased approaches to sample analysis in tumours, from quality control to biomarker validation and clinical annotation. The high dimensionality of collated imaging data, and in particular the skewed distributions of biomarker image features, does require novel analysis tools such as RSF [49]. Moreover, new strategies in development that aim to reduce dimensionality, and so minimise the risk of over fitting when using random forest, and ultimately the performance of these approaches, will likely redefine the concept of sample size in biomedical applications [67]. As the subsets of the genomic enriched classification of cancer further sub-divide, rigid application of the ‘test-validation’ cohorts as developed in biomarker guidelines such as REMARK are unlikely to be helpful longer term, especially if data is directly linked to genomic analysis [68], [69]. In particular, the quality assurance at the molecular level will be essential as a first analysis before any prospect of useful cross-validation in independent cohorts can be attempted, even if sample processing guidelines are followed, e.g. BRISQ guidelines. Thus to maximise the informative and predictive capabilities provided by unbiased machine learning algorithms such as RF, the standardisation of protocols for collecting and processing high quality material are still required [25].

A further application of our methodology of single cell segmentation and biomarker distribution could equally apply to determining the response to agents and drugs. The relative changes between multiple small core biopsies before and after exposure to agents may identify cell populations resistant to target inhibition. We are currently applying our methodology in a Ewing sarcoma exploratory biomarker Phase II study (LINES trial, EuroSarc) in patients with advanced disease. The drug tested in this case is linsitinib, a dual IGF1R/IR-A kinase inhibitor.

In summary, we combined single cell imaging data from tissue into a high dimensional feature distribution and a cross-validated RSF to generate a pipeline for discovery of prognostic classifiers (summarised in Figure 3). Importantly, such unbiased analysis can lead to the generation of new hypotheses requiring testing, and in particular relating to sub-populations of cells in a tumour that may have a disproportionate contribution to clinical outcomes in genetically characterised cancer sample cohorts.

## Supporting Information

Table S1 **Ewing sarcoma cell lines (EuroBoNeT).**
(DOCX)

Table S2 **Validation data of antibodies utilised.**
(DOCX)

Table S3 **Outcome data of Ewing sarcoma patients with good and poor images.**
(XLSX)

File S1 **Figures S1–S13.** Figure S1. Image segmentation algorithm (*OxBioPath*- segmentation.v1). Figure S2. Heterogeneity of signalling responses quantified in Ewing cell lines. Figure S3. Cumulative frequency plots of the Ewing sarcoma signalling biomarkers. Figure S4. Heterogeneity of signalling pathway activity in Ewing cell line cores. Figure S5. Image quality control criteria. Figure S6. Patient survival comparison between imaged and non-imaged cohort data. Figure S7. Automated quality control classifier for DAPI images. Figure S8. Visualising the distribution of cell features with principal components analysis. Figure S9 Density plots of mean nuclear vs. mean cytoplasmic CD99 and Ki67 in each segmented cell across all imaged patients and cohorts. Figure S10, S11 and S12. Predicted relative mortality and survival plots from random survival forest cross-validation. Figure S13. Example of segmented tumour cell images showing Ki67 positive CD99 negative (high nuclear: cytoplasmic ratio) cells.
(PDF)
