# Supplementary material for: Quantification of the Heterogeneity of Prognostic Cellular Biomarkers in Ewing Sarcoma Using Automated Image and Random Survival Forest Analysis
Source: PLoS One. 2014 Sep 22;9(9):e107105. doi: 10.1371/journal.pone.0107105 (PMC4171480; doi:10.1371/journal.pone.0107105)
Supplement: Table S1 — Ewing sarcoma cell lines (EuroBoNeT). (DOCX) [file pone.0107105.s001.docx]

**Supporting Information**

**Table S1**

**Quantification of the heterogeneity of prognostic cellular biomarkers in Ewing sarcoma using automated image and random survival forest analysis**

Claudia Bühnemann^1*^, Simon Li^2*^, Haiyue Yu^1,2^, Harriet Branford White^1^, Karl L. Schäfer^3^, Antonio Llombart-Bosch^4^, Isidro Machado^4^, Piero Picci^5^, Pancras C.W. Hogendoorn^6^, Nicholas A. Athanasou^7^, J.Alison Noble^2^, A. Bassim Hassan^1¶^

**Table S1 Ewing sarcoma cell lines (EuroBoNeT)**

| **Cell Line** | **Diagnosis** | **Source** | **Age** | **Sex** | **Translocation** | **Type of Fusion** | **p53** |
| --- | --- | --- | --- | --- | --- | --- | --- |
| **A673*** | ES | Muscle | 15 | F | t(11;22) | 7/6 | c.551_552insCA |
| **CHP-100** | pPNET | Mediastinum | 12 | F | t(11;22) | _ | wt |
| **RD-ES** | ES | Humerus | 19 | M | t(11;22) | 7/5 | p.Arg273Cys |
| **RM-82** | ES | Femur | 8 | M | t(21;22) | 7/6 | p.Arg273His |
| **SK-N-MC** | pPNET | Supraorbital | 14 | F | t(11;22) | _ | c.170_572del |
| **STA-ET1** | pPNET | Humerus | 13 | F | t(11;22) | _ | wt |
| **STA-ET2.1** | pPNET | Fibula | 15 | M | t(11;22) | _ | p.Cys277tyr |
| **STA -ET10** | ES | Muscle (paraspinal) | 2 | F | t(2;22) | _ | wt |
| **TC71** | ES | Humerus | 22 | M | t(11;22) | 7/5 | p.Arg213X |
| **WE-68** | ES | Fibula | 19 | F | t(11;22) | 7/6 | wt |

*B-Raf mutation (V600E)

7/5 fusion refers to exon 7 of EWS fused to exon 5 of FLI1
