# Supplementary material for: Quantification of the Heterogeneity of Prognostic Cellular Biomarkers in Ewing Sarcoma Using Automated Image and Random Survival Forest Analysis
Source: PLoS One. 2014 Sep 22;9(9):e107105. doi: 10.1371/journal.pone.0107105 (PMC4171480; doi:10.1371/journal.pone.0107105)
Supplement: Table S2 — Validation data of antibodies utilised. (DOCX) [file pone.0107105.s002.docx]

**Supporting Information**

**Table S2**

**Quantification of the heterogeneity of prognostic cellular biomarkers in Ewing sarcoma using automated image and random survival forest analysis**

Claudia Bühnemann^1*^, Simon Li^2*^, Haiyue Yu^1,2^, Harriet Branford White^1^, Karl L. Schäfer^3^, Antonio Llombart-Bosch^4^, Isidro Machado^4^, Piero Picci^5^, Pancras C.W. Hogendoorn^6^, Nicholas A. Athanasou^7^, J.Alison Noble^2^, A. Bassim Hassan^1¶^

**Table S2 Validation data of antibodies utilised**

| **Antibody Registry No.**  **(all rabbit mAb except*)** | **Antigen** | **Sensitivity and Specificity** | **Application on ES cell lines (IF) or tumours (IHC)** | **Validated tissue** | **Reference** |
| --- | --- | --- | --- | --- | --- |
| [AB_149707](http://antibodyregistry.org/AB_149707)  Ki67, clone SP6,  (#RM-9106),  Thermo Scientific | Synthetic peptide to C-terminus human Ki67 | Labelling in proliferating tissues only | IHC | Murine mammary tumours  Murine bladder cancer  Human glioblastoma xenografts  Human colorectal cancer | Louie, K.B. et al. (2013) [Sci Rep](http://www.ncbi.nlm.nih.gov/pubmed/23584513?dopt=Abstract) 3 (3) 1656, doi:10.1038/srep01656*****  Hurst R.E. et al. (2013) Plos one, 8 (5), e64181. doi:10.1371/journal.pone.0064181*****  Viel, T. et al. (2013) Plos one 8 (7) e67911. doi: 10.1371/journal.pone.0067911*****  Fodor I.K., et al. (2012) J Clin Pathol 65, 989 - 995 |
| [AB_2097035](http://antibodyregistry.org/AB_2097035)  Egr1, clone 44D5  (#4154), Cell signaling | Synthetic peptide to N-terminus human EGR1 | NGF dependent single band western blot, flow cytometry and localisation change | IF  IHC | Pheochromocytoma tumour from the rat adrenal medulla  Dopaminergic neurons in rat brains | According to data sheet from Cell signaling  Day, J.J. et al. (2013) Nat Neurosci 16, 1445-1452 |
| [AB_836876](http://antibodyregistry.org/AB_836876)  FoxO3a, clone 75D8 (#2497), Cell signaling | Synthetic peptide around Glu50 human Foxo3a | Single band Western blot absent in Foxo3a knockout brain, localisation change to LY294002  confocal | IF | Human neuroblastoma cell line (SH-SY5Y)  Human mononuclear bone marrow leukemia cells | According to data sheet from Cell signaling  Sykes, S.M. et al. (2001) Cell 146, 697-708 |
| [AB_2106672](http://antibodyregistry.org/AB_2106672)  FoxO3a, rabbit polyclonal* (#9467), Cell signaling | Synthetic peptide around Glu50 human Foxo3a | Single band western blot, labelling of tissues | IHC | Colorectal Cancer | Bullock M.D. et al. (2013) BJC 109, 387-394 |
| [AB_2181035](http://antibodyregistry.org/AB_2181035)Phospho-S6, clone 91B2 (#4857), Cell signaling | Synthetic phospho peptide Ser 235/Ser236 of human S6 | Western single band, inhibition by rapamycin, tissue cytoplasmic labelling | IF | Murine dopaminergic neurons  Hela cells | Kim T.W. et al. (2013) [Cell Death Dis](http://www.ncbi.nlm.nih.gov/pubmed/?term=Kim+T.W.%2C+%282013%29%2C+Cell+Death+Dis+4%2C+%2C+e919%3B+doi%3A10.1038%2Fcddis.2013.447), 14, e919; doi:10.1038/cddis.2013.447  Hofman, G.R. et al. (2010) Assay Drug Dev Technol, 8(2), 186–199 |
| [AB_916160](http://antibodyregistry.org/AB_916160)Phospho-S6 Alexa647, clone D57.2.2E (#4851), Cell signalling | Synthetic phospho peptide Ser 235/Ser236 of human S6 | Flow cytometry, rapamycin and IF in tissue sections | IHC | Paraffin-embedded H3255 (human non-small cell lung cancer cell line) xenograft | According to data sheet from Cell signaling |
| [AB_2315112](http://antibodyregistry.org/AB_2315112)  pERK1/2, clone D13.14.4E (#4370), Cell signalling | Synthetic phospho- peptide Thr202/ Tyr 204 of human p44 MAPK | UO126 dependent labelling, Western blots, | IHC | Human mammary xenografts  Murine kidney cancer  Murine lung cancer | Faber, A. et al. (2011) Cancer Discov 1, 352-365  Hudon, V. et al. (2010) J Genet 47, 182-9  Engelman, J.A. (2008) Nat Med 14, 1351-6 |

- Immunohistochemical labelling with Ki67 in these publications was performed with the same clone (SP6) but the antibody was purchased from Abcam and not from Thermo scientific.
- Antibody Registry code from; http://antibodyregistry.org
