# Supplementary material for: Quantification of the Heterogeneity of Prognostic Cellular Biomarkers in Ewing Sarcoma Using Automated Image and Random Survival Forest Analysis
Source: PLoS One. 2014 Sep 22;9(9):e107105. doi: 10.1371/journal.pone.0107105 (PMC4171480; doi:10.1371/journal.pone.0107105)
Supplement: File S1 — Figures S1–S13. Figure S1. Image segmentation algorithm (OxBioPath- segmentation.v1). Figure S2. Heterogeneity of signalling responses quantified in Ewing cell lines. Figure S3. Cumulative frequency plots of the Ewing sarcoma signalling biomarkers. Figure S4. Heterogeneity of signalling pathway activity in Ewing cell line cores. Figure S5. Image quality control criteria. Figure S6. Patient survival comparison between imaged and non-imaged cohort data. Figure S7. Automated quality control classifier for DAPI images. Figure S8. Visualising the distribution of cell features with principal components analysis. Figure S9 Density plots of mean nuclear vs. mean cytoplasmic CD99 and Ki67 in each segmented cell across all imaged patients and cohorts. Figure S10, S11 and S12. Predicted relative mortality and survival plots from random survival forest cross-validation. Figure S13. Example of segmented tumour cell images showing Ki67 positive CD99 negative (high nuclear: cytoplasmic ratio) cells. (PDF) [file pone.0107105.s004.pdf]

Supporting Information
Figures S1-S13

Quantification of the heterogeneity of prognostic cellular biomarkers in Ewing sarcoma using automated image and random survival forest analysis

Claudia Bühnemann[1*], Simon Li[2*], Haiyue Yu[1,2], Harriet Branford White[1], Karl L. Schäfer[3], Antonio Llombart-Bosch[4], Isidro Machado[4], Piero Picci[5], Pancras C.W. Hogendoorn[6], Nicholas A. Athanasou[7], J.Alison Noble[2], A. Bassim Hassan[1¶]

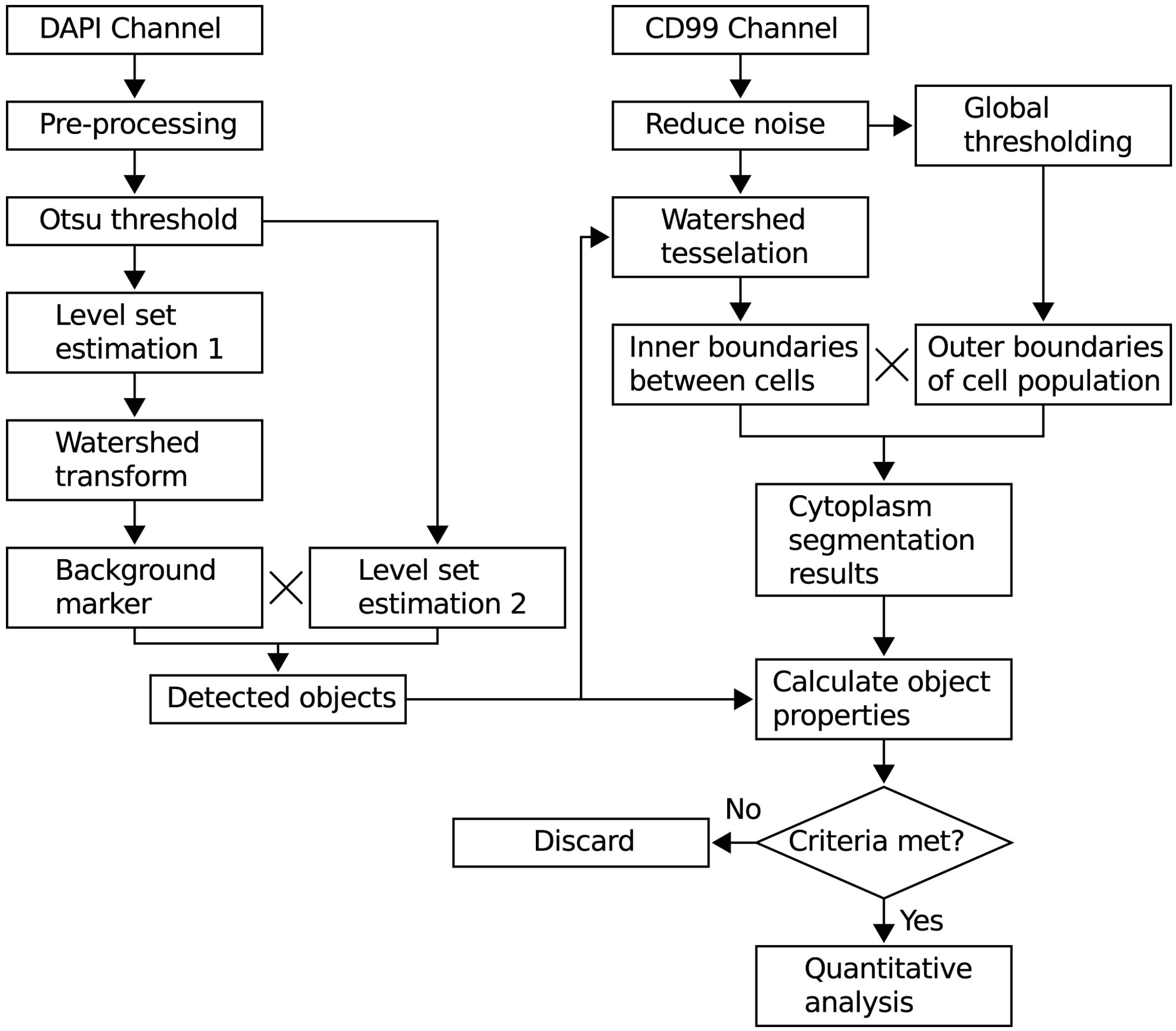

Figure S1.
Image segmentation algorithm (*OxBioPath*- segmentation.v1)
For segmentation of the nucleus and cytoplasm, DAPI and cytoplasmic channels (CD99 or phalloidin) were processed by starting with an Otsu threshold to initiate a level set segmentation. A combination of global thresholding and watershed tessellation derived from segmentation of nuclei was combined. A simple quality control criteria based on the area of the nucleus (<500 pixels) was then determined. If small particles were therefore excluded, the nuclei informative for further analysis were all >500 pixels (µM) .

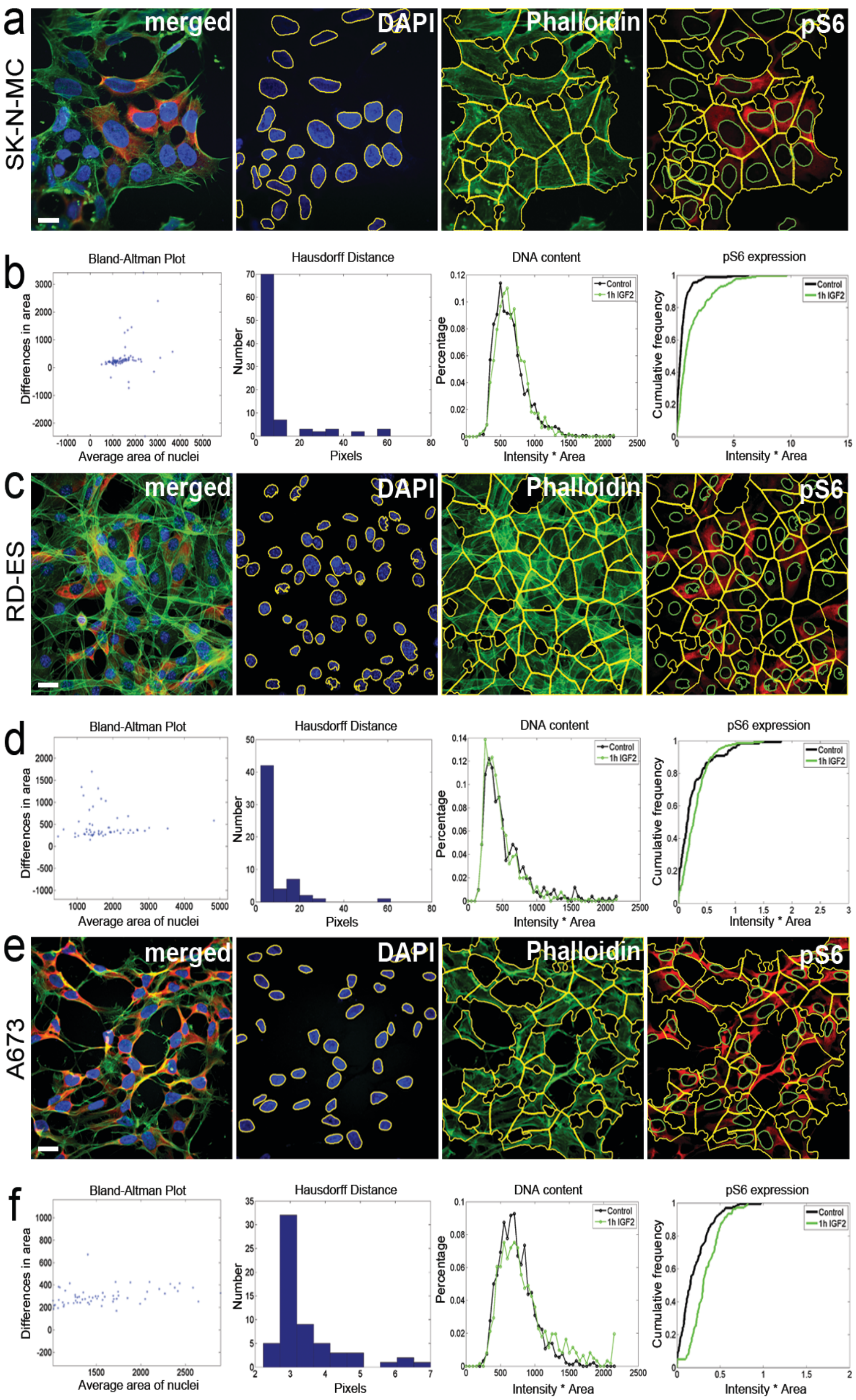

Figure S2.

Heterogeneity of signalling responses quantified in Ewing cell lines.

Signalling responses were quantified after treatment with IGF2. **a**. **b**. SK-N-MC, **c**. **d**. RD-ES and **e**. **f**. A673 Ewing sarcoma cell lines were incubated with IGF2 for 1h after 24h serum starvation. The images were captured with confocal microscopy and segmented as in Supplementary Fig. 1, with a combination of nuclear (DAPI) and cytoplasmic mask (in this case Phalloidin,). The activation of the IGF pathway is revealed by an increase in the expression intensity of the ribosomal protein pS6 in the cytoplasm, with distribution of nuclear DNA and pS6 shown with Bland-Altman and Hausdorff validation. Bar 10μM.

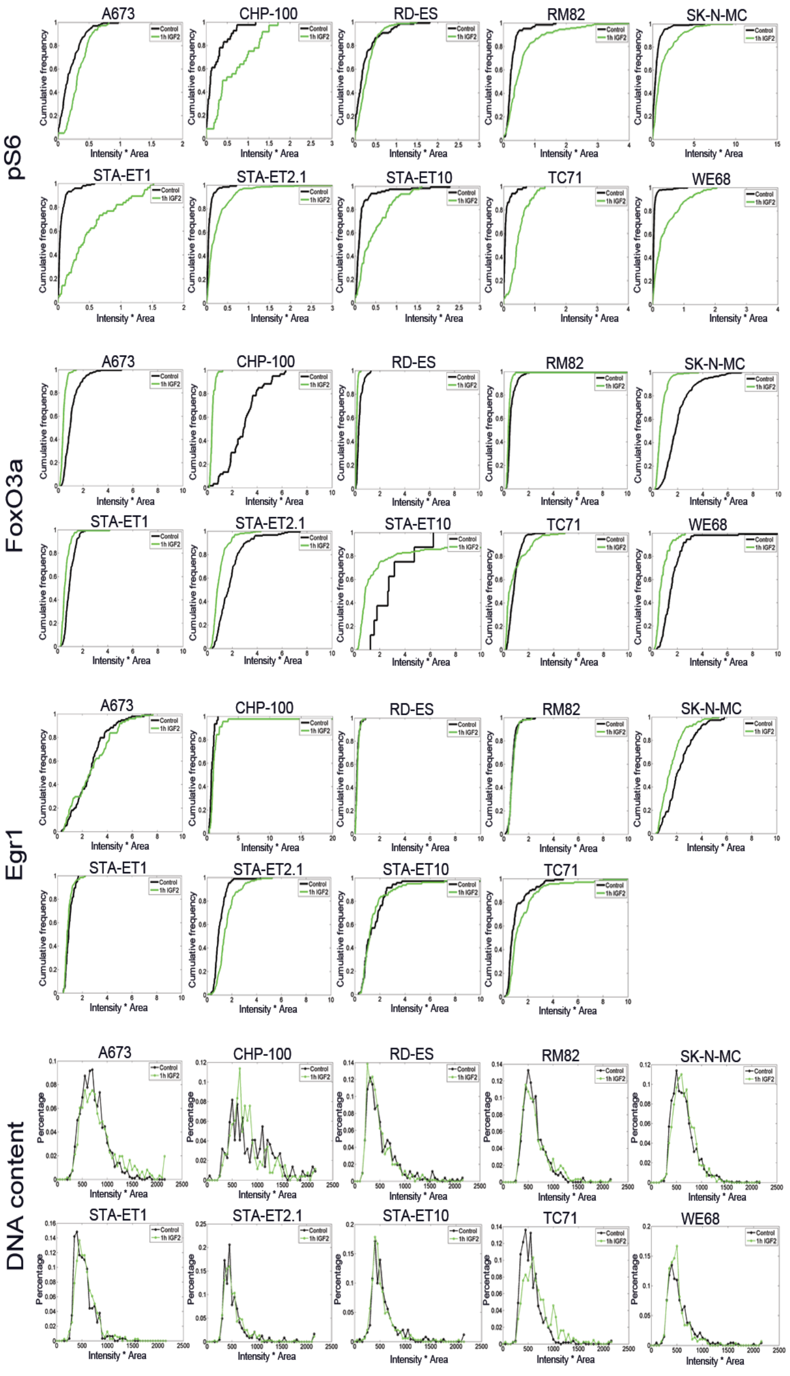

Figure S3.

Cumulative frequency plots of the Ewing sarcoma signalling biomarkers.

Signalling pathway activity was quantified with image analysis as in Supplementary Figures 1 and 2. pS6 (cytoplasmic), Foxo3a (nuclear) and EGR1 (nuclear) in Ewing sarcoma cell lines were quantified in each cell compartment before and after treatment with the ligand IGF2. Each cell line responded differently and with different degrees of heterogeneity. Total DNA intensity of both control and IGF2 treated group showed similarity within the cell lines. These data indicate the utility of the segmentation algorithm in the detection of biomarker heterogeneity.

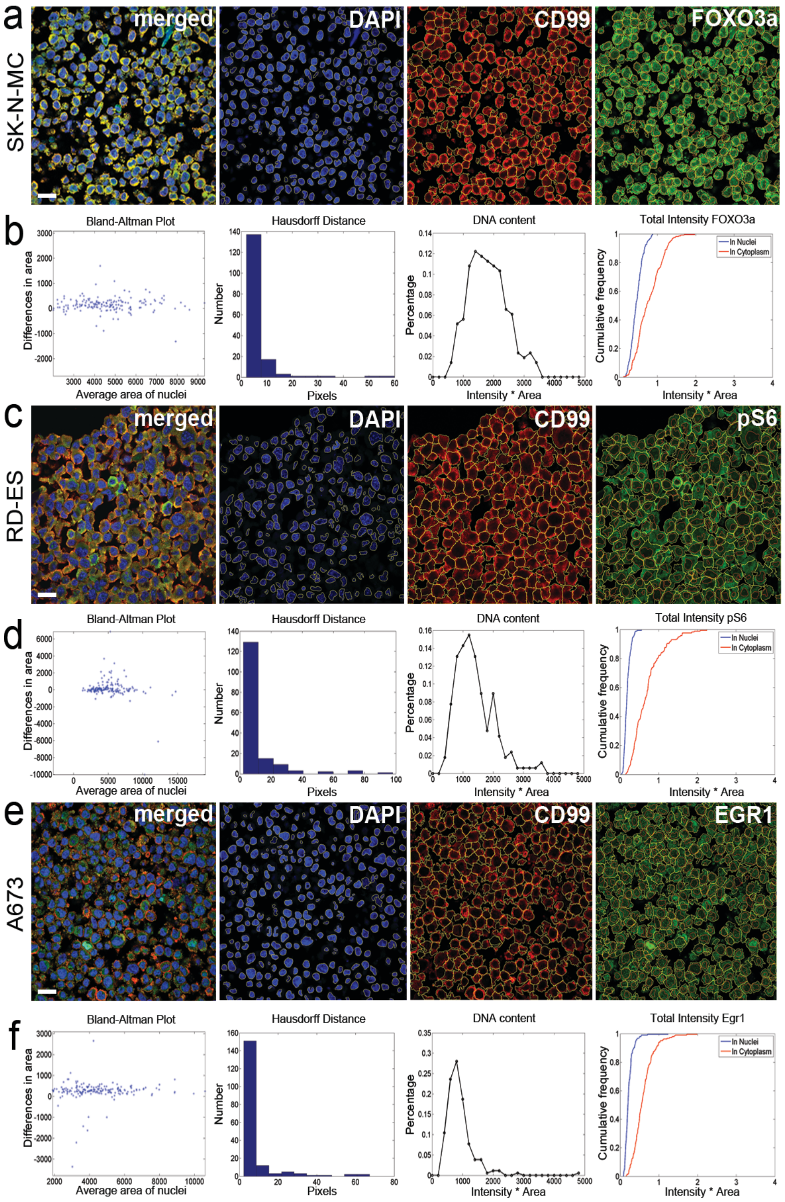

Figure S4.

Heterogeneity of signalling pathway activity in Ewing cell line cores

Signalling pathway activity was quantified in agarose cell pellets of Ewing sarcoma cell lines SK-NMC (**a**., **b**.), RD-ES (**c**., **d**.), and A673 (**e**., **f.**) in order to mimic tumour cell overlap and clumping. The images were captured by confocal microscopy and segmented using nuclear DAPI and cytoplasmic CD99 (**a**., **c**., **e**.). The activation of the signalling pathway was revealed by the altered nuclear and cytoplasmic intensity of Foxo3a (**a**.) and EGR1, (**e.**) the cytoplasmic ribosomal protein pS6 (**c**.), and distribution of nuclear DNA (DAPI) (**a**., **c**., **e**.). Segmentations for each data set were compared with manual segmentations using Bland-Altman and Hausdorff plots. These data resulted in changes to the parameters of the segmentation algorithm used on tissue microarray samples (Fig. 1). Bar 10μM

Image quality control

No.	Criteria	Example
1	There are less than 10% of the cells expressing CD99 in membrane and/or cytoplasm.	
2	There are less than 50 cells per image.	
3	The image contains more than 10% stromal tissue.	
4	There are less than 20% of the nuclei having clear defined boundaries.	
5	There are more than 30% of the nuclei in a clump that prevents resolution of cellular boundaries.	

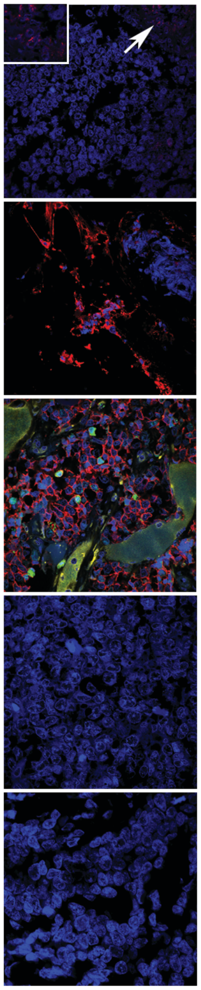

Figure S5. Image quality control criteria
The quality of the tissue microarray material was highly variable and poor quality images would obscure the information contained in higher quality images. To exclude uninformative images, five criteria were defined; lack of CD99 labelling (cytoplasmic mask) leading to a lack of cytoplasmic segmentation, low number of cells, significant proportion of stromal tissue to tumour tissue, poor DNA quality and unable to segment DAPI images, with disruption in DNA nuclear boundaries and associated smearing, and a large proportion of the image comprised overlapping cells in clumps that was also impossible to segment. For the majority of samples (62%), the quality of the cores on the TMA slides resulted in omission of patients from subsequent analysis (see Fig. 6). Note that we discard cells with area (pixels) which do not lie within the following bounds: nucleus <1500 nucleus > 8000: <1000 cytoplasm >25000.

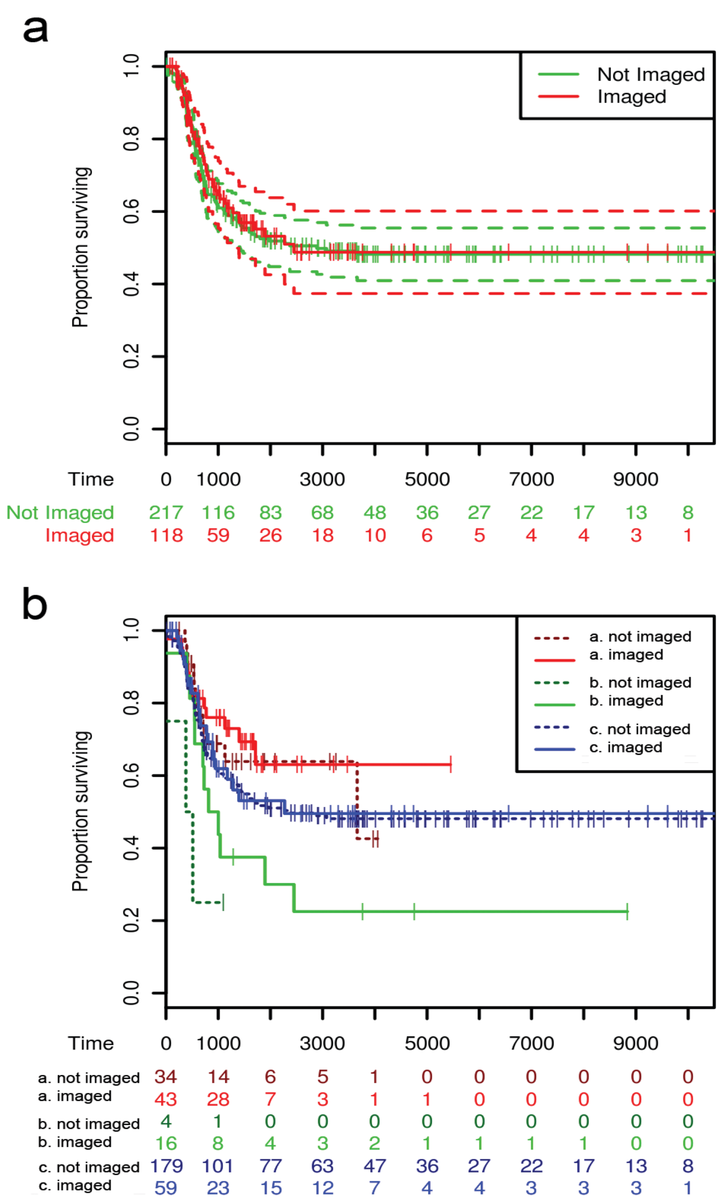

Figure S6.

Patient survival comparison between imaged and non-imaged cohort data.

As a result of image quality control criteria two groups of samples were obtained, images that proceeded to full analysis (informative or imaged) and those that did not proceed and were not imaged (uninformative or image capture but not full segmentation and image analysis). **a**. Overall survival of imaged and not imaged samples with 95% confidence intervals (dashed lines), and **b**. comparison of imaged and not imaged samples by cohort. These data show that the imaged samples were representative of the overall cohort, and within each cohort, as judged by survival. The majority of the samples of lower quality were in cohort ‘c’.

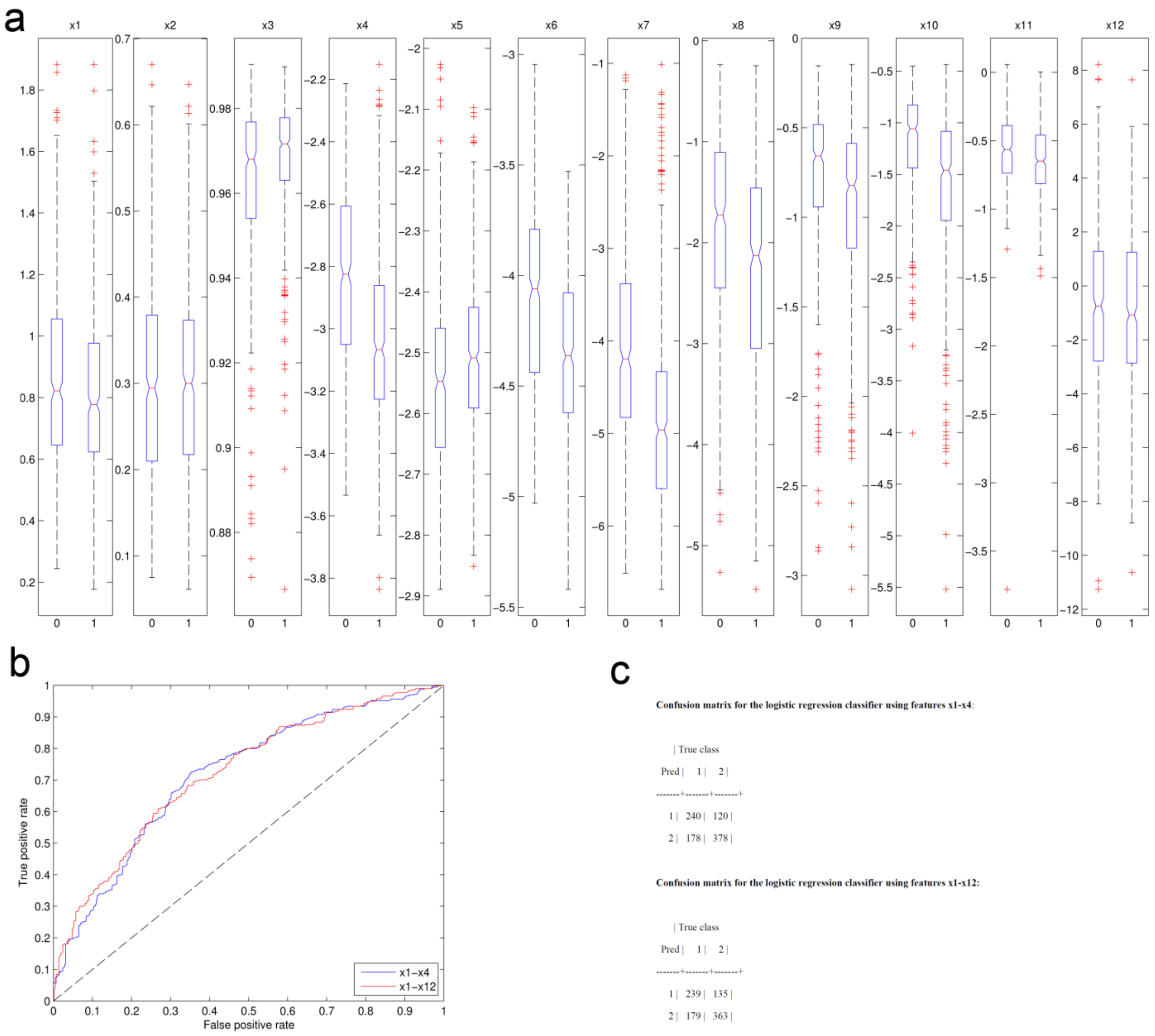

Confusion matrix for the logistic regression classifier using features x1-x4:

Pred	True class 1	True class 2
1	240	120
2	178	378

Confusion matrix for the logistic regression classifier using features x1-x12:

Pred	True class 1	True class 2
1	239	135
2	179	363

Figure S7. Automated quality control classifier for DAPI images

In order to support future automation of the entire pipeline we built an automated classifier using a set of image features designed for high content screening using the DAPI channel from images also stained for Ki67 (916 images). 230 of these images were labelled as poor quality and 686 as good quality. Four HCS QC features were calculated for each image. **a.** The distribution of each calculated HCS feature is summarised for poor and good quality DAPI (Ki67 dataset) images that passed (1) or failed (0) the manual quality control stage. x1-x4 are HCS quality control features, x5-x12 are sub-components of x4. **b.** Leave-one-out cross validation ROC curves for logistic regression classifiers using either four or twelve HCS features. Area under curve: x1-x4=0.72, x1-x12=0.72. Receiver operating characteristic curves are shown for both classifiers, in both cases the area under curve was the same indicating there was no benefit to including the additional features. **c**. Confusion matrix for the logistic regression classifier using feature x1-x4 and x1-x12.

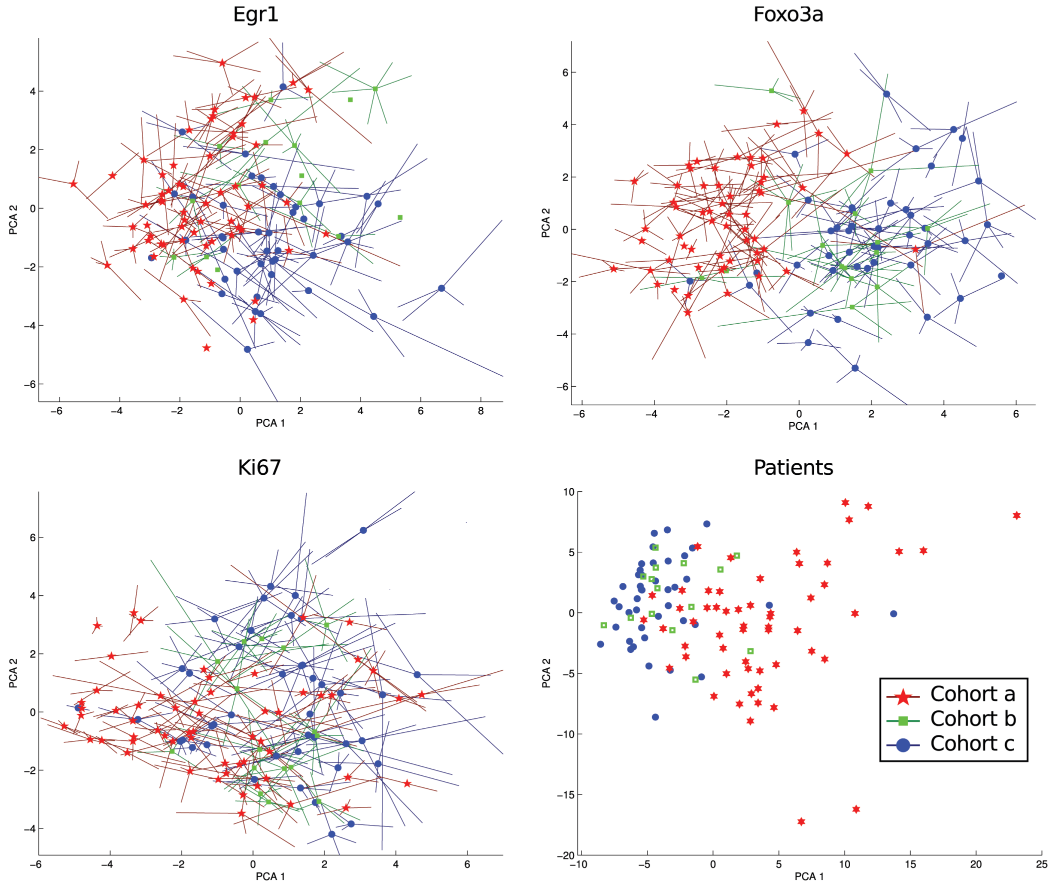

Figure S8.

Visualising the distribution of cell features with principal components analysis.

The first two principal components for each patient are shown for biomarkers EGR11, Foxo3a and Ki67. These are 2D plots in which the central dot (with respect to cohort) is the mean and the projections are the individual values for each image of that patient. Thus each patient's separate image data, although displaying within patient variation, tended to cluster within the PCA space more closely compared to images from different patients. Scatter plot of the two principal components of patient level features is also shown. In order to combine data from each cohort, one needs to make the assumption that all images from a single patient are drawn form the same distribution in order to combine the three datasets into one. If PCA is performed on these patient-level features we start to see evidence of a systematic difference between the datasets, which may relate to the fundamental differences in survival of the patients in each dataset or to differences in pre-processing.

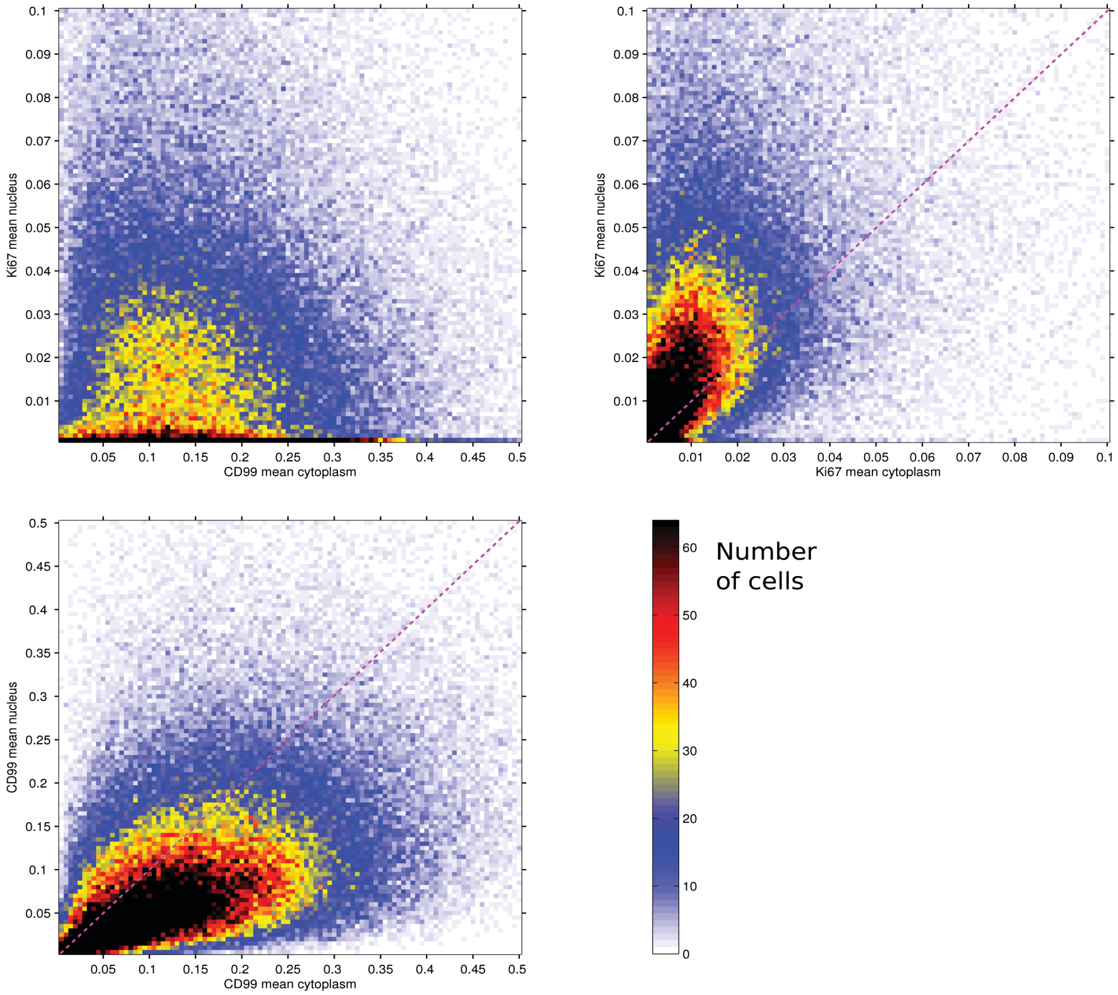

Figure S9.

Density plots of mean nuclear vs. mean cytoplasmic CD99 and Ki67 in each segmented cell across all imaged patients and cohorts.

The magenta dotted line of equality represents the division of cells into two groups based on whether the mean nuclear intensity is either greater than or less than the mean cytoplasmic intensity in each cell. From these distributions, a proportion of cells have Ki67 levels that are greater in the cytoplasm than in the nucleus (Ki67 negative), and a group of cells that have greater CD99 levels in the nucleus than the cytoplasm (CD99 negative).

Figure S10, S11 and S12.
Predicted relative mortality and survival plots from random survival forest cross-validation.
Predicted relative mortality (vertical axis) compared with actual survival time (horizontal axis, truncated at 2500 days) for the test set in 25 cross validation repeats of the RSF of nuclear/ cytoplasmic marker ratios in CD99 positive and negative cells with Ki67 positive (CD99 all Ki67, **Fig. 10a**), CD99 positive with Ki67 (CD99+Ki67, **Fig. 11a**) and CD99 negative with Ki67 positive (CD99-Ki67, **Fig. 12a**). All 50 repeats were sorted by increasing error rate, and plots for alternate repeats are shown. Red cross: deceased, Black dot: censored, error rates shown in blue. Survival curves using real data (solid lines) and individual patient predictions (dashed lines) for the test set in 25 cross-validation repeats of the CD99 all Ki67 (**Fig. 10b**), CD99+Ki67 (**Fig. 11b)** and CD99-Ki67 (**Fig. 12b**) corresponding to part **a**. Patients were split into two groups using the RSF predicted mortality (blue: low, red: high), with the more convincing separation evident for CD99-Ki67 (**Fig 12a and b**).

Figure S10

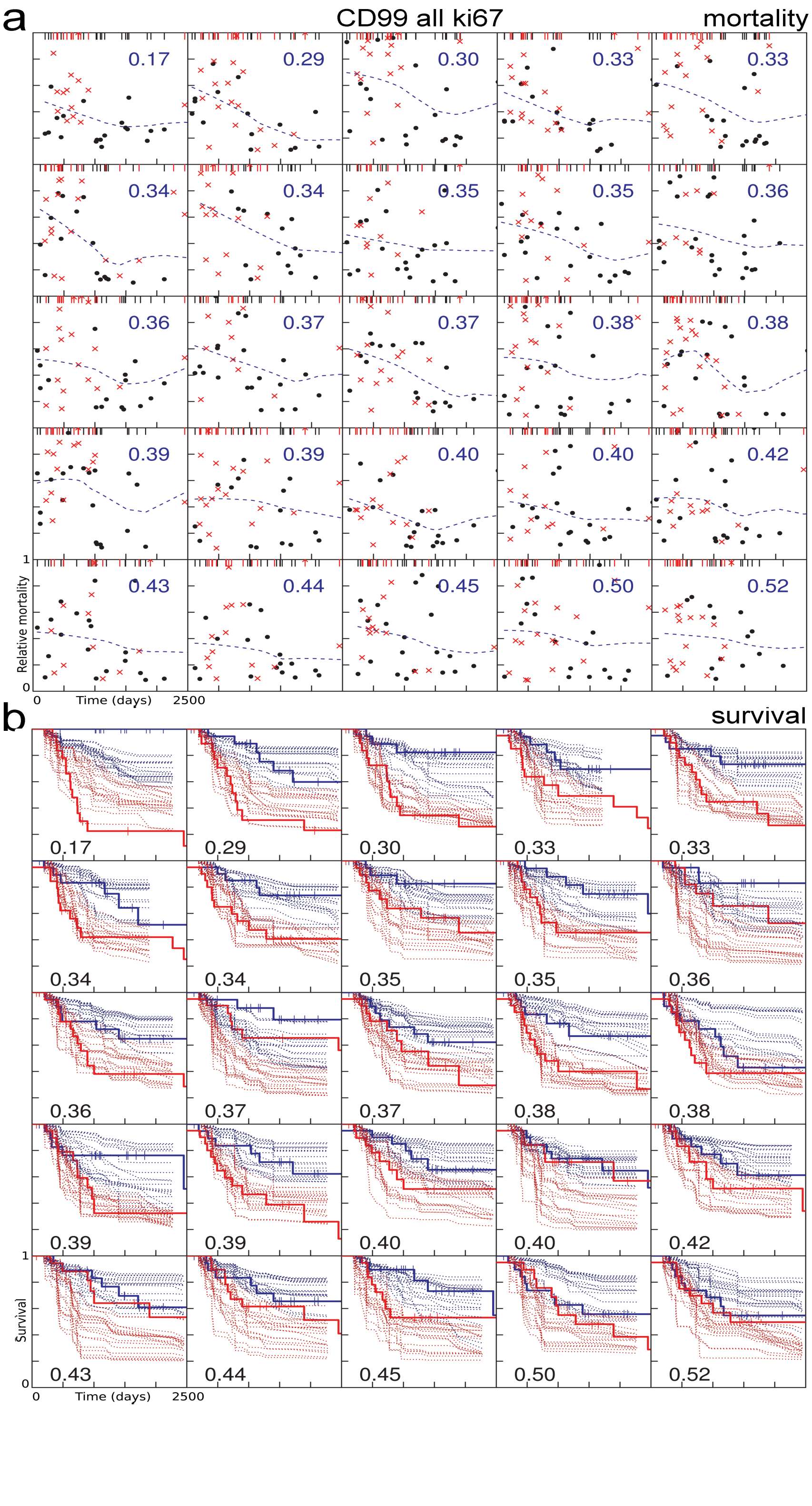
a
CD99 all ki67
mortality
0.17
0.29
0.30
0.33
0.33
0.34
0.34
0.35
0.35
0.36
0.36
0.37
0.37
0.38
0.38
0.39
0.39
0.40
0.40
0.42
0.43
0.44
0.45
0.50
0.52
Relative mortality
1
0
0
Time (days)
2500
b
survival
0.17
0.29
0.30
0.33
0.33
0.34
0.34
0.35
0.35
0.36
0.36
0.37
0.37
0.38
0.38
0.39
0.39
0.40
0.40
0.42
0.43
0.44
0.45
0.50
0.52
Survival
1
0
0
Time (days)
2500

Figure S11

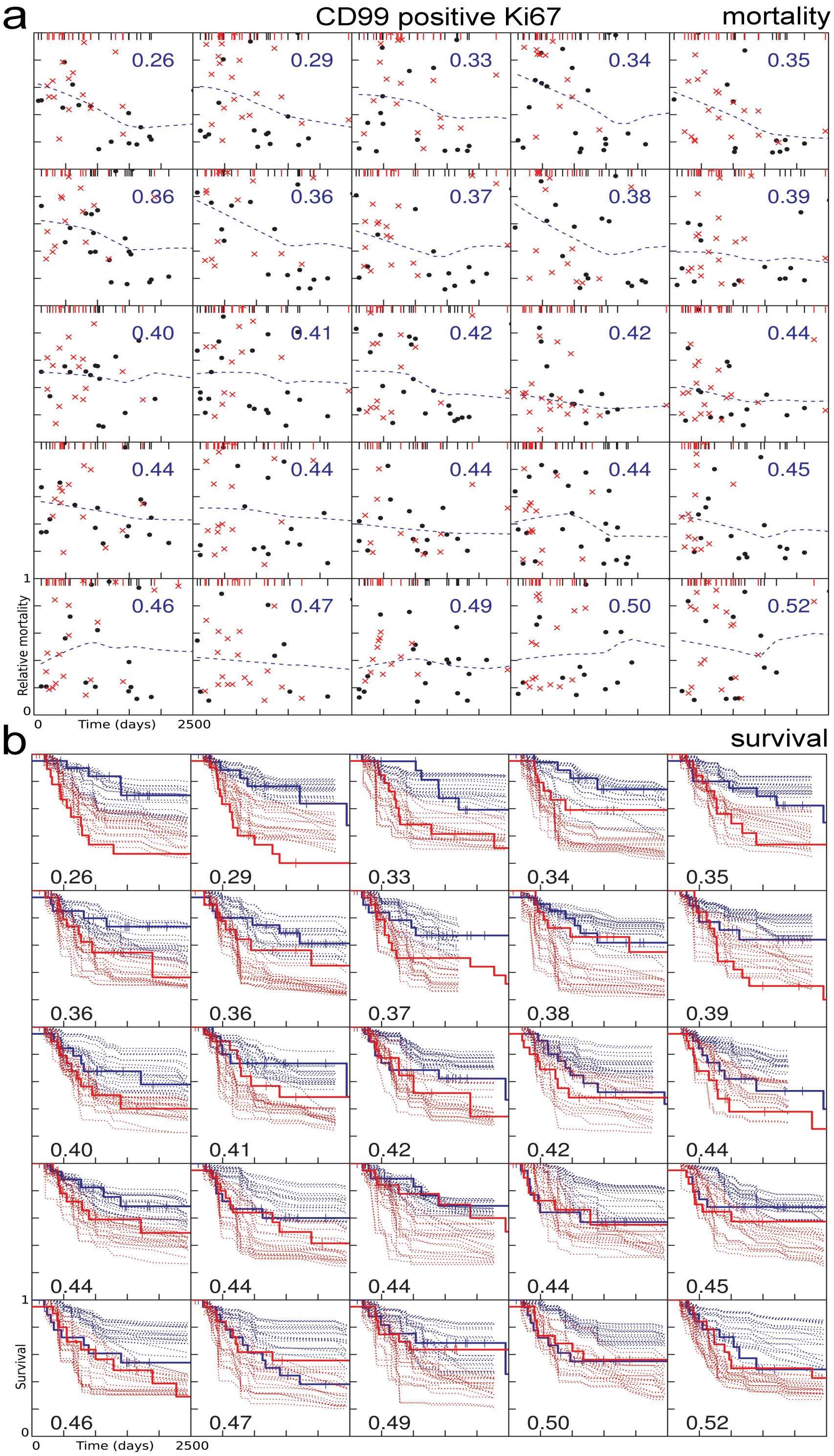
a
CD99 positive Ki67
mortality
0.26
0.29
0.33
0.34
0.35
0.36
0.36
0.37
0.38
0.39
0.40
0.41
0.42
0.42
0.44
0.44
0.44
0.44
0.44
0.45
0.46
0.47
0.49
0.50
0.52
Relative mortality
1
0
0
Time (days)
2500
b
survival
0.26
0.29
0.33
0.34
0.35
0.36
0.36
0.37
0.38
0.39
0.40
0.41
0.42
0.42
0.44
0.44
0.44
0.44
0.44
0.45
0.46
0.47
0.49
0.50
0.52
Survival
1
0
0
Time (days)
2500

Figure S12

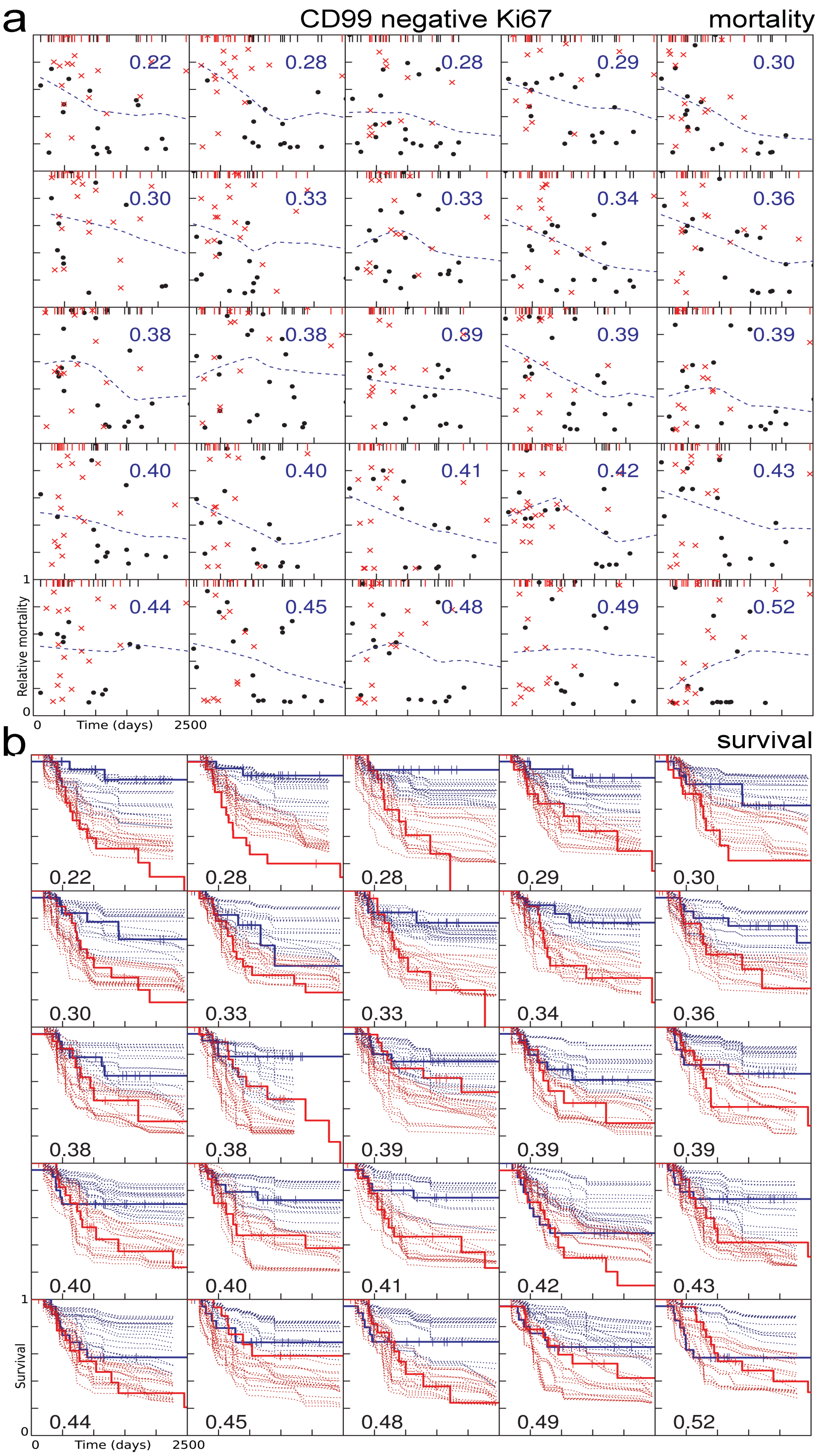
a
CD99 negative Ki67
mortality
0.22
0.28
0.28
0.29
0.30
0.30
0.33
0.33
0.34
0.36
0.38
0.38
0.39
0.39
0.39
0.40
0.40
0.41
0.42
0.43
0.44
0.45
0.48
0.49
0.52
Relative mortality
1
0
0
Time (days)
2500
b
survival
Survival

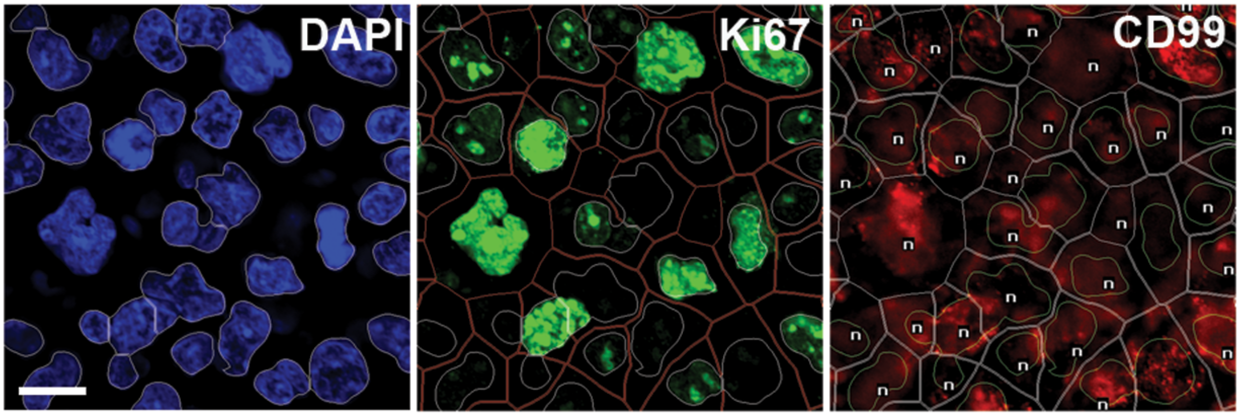

Figure S13.
Example of segmented tumour cell images showing Ki67 positive CD99 negative (high nuclear: cytoplasmic ratio) cells. Segmentation based on DAPI and CD99 identified Ki67 positive cells that had high nuclear and low cytoplasmic CD99 (referred to as CD99 negative as per Supplementary Fig 9). RSF with cross-validation identified this population as prognostic in Ewing sarcoma. Bar 10μm.
